# Transcriptome Analysis and Gene Identification in the Pulmonary Artery of Broilers with Ascites Syndrome

**DOI:** 10.1371/journal.pone.0156045

**Published:** 2016-06-08

**Authors:** Fei Yang, Huabin Cao, Qingyang Xiao, Xiaoquan Guo, Yu Zhuang, Caiying Zhang, Tiancheng Wang, Huayuan Lin, Yalu Song, Guoliang Hu, Ping Liu

**Affiliations:** Institute of Animal Population Health, College of Animal Science and Technology, JiangXi Agriculture University, N.O. 1101, Zhimin Avenue, Nanchang Economic and Technological Development District Nanchang, 330045, P. R. China; Vanderbilt University Medical Center, UNITED STATES

## Abstract

**Background:**

Pulmonary arterial hypertension, also known as Ascites syndrome (AS), remains a clinically challenging disease with a large impact on both humans and broiler chickens. Pulmonary arterial remodeling presents a key step in the development of AS. The precise molecular mechanism of pulmonary artery remodeling regulating AS progression remains unclear.

**Methodology/Principal Findings:**

We obtained pulmonary arteries from two positive AS and two normal broilers for RNA sequencing (RNA-seq) analysis and pathological observation. RNA-seq analysis revealed a total of 895 significantly differentially expressed genes (DEGs) with 437 up-regulated and 458 down-regulated genes, which were significantly enriched to 12 GO (Gene Ontology) terms and 4 KEGG (Kyoto Encyclopedia of Genes and Genomes) pathways (Padj<0.05) regulating pulmonary artery remodeling and consequently occurrence of AS. These GO terms and pathways include ribosome, Jak-STAT and NOD-like receptor signaling pathways which regulate pulmonary artery remodeling through vascular smooth cell proliferation, inflammation and vascular smooth cell proliferation together. Some notable DEGs within these pathways included downregulation of genes like RPL 5, 7, 8, 9, 14; upregulation of genes such as IL-6, K60, STAT3, STAT5 Pim1 and SOCS3; IKKα, IkB, P38, five cytokines IL-6, IL8, IL-1β, IL-18, and MIP-1β. Six important regulators of pulmonary artery vascular remodeling and construction like CYP1B1, ALDH7A1, MYLK, CAMK4, BMP7 and INOS were upregulated in the pulmonary artery of AS broilers. The pathology results showed that the pulmonary artery had remodeled and become thicker in the disease group.

**Conclusions/Significance:**

Our present data suggested some specific components of the complex molecular circuitry regulating pulmonary arterial remodeling underlying AS progression in broilers. We revealed some valuable candidate genes and pathways that involved in pulmonary artery remodeling further contributing to the AS progression.

## Introduction

Ascites syndrome (AS, pulmonary artery hypertension-PAH), a metabolic disorder frequently observed in broiler chickens, is primarily the result of elevated pressure in the pulmonary artery [1–3]. If the pulmonary vasculature is continually engorged by fast blood flow to the extent that it becomes non-expandable, the circulation hemodynamic blood pressure in pulmonary vessels will increase, leading to pulmonary artery hypertension. To propel excessive blood flow throughout the lungs, the right ventricle is subjected to higher pressures, further resulting in right ventricular hypertrophy and failure [4]. Many previous studies have confirmed that rapid growth, low temperature, and a high energy diet increase the metabolic demand for oxygen, resulting in internal hypoxia and further stimulating excessive output from the heart and influx into the pulmonary artery, a clear factor involved in the occurrence of PAH [5–7]. Furthermore, broilers with AS were found to have higher pulmonary artery pressure and pulmonary vasculature resistance to blood flow [3]. Indeed, any factor inducing prolonged pulmonary vasoconstriction and vascular obstruction that leads to a reduction in pulmonary vascular volume could elevate vascular resistance, thereby exacerbating the development of AS [7]. By pathological structural observations, the pulmonary artery has been shown to be remodeled and the thickness of vascular smooth muscle increased, leading to a decrease in arterial radius [8] and causing an increase in the resistance to blood flow. Thus, changes in the pulmonary artery are closely related to the pathogenesis of ascites syndrome.

Recently, the investigation of pulmonary artery remodeling pathogenesis during AS progression on the molecular level has caused much attention from researchers. Calcium signaling was found to be involved in the development of PAH in broilers, and calcium antagonists such as verapamil, diltiazem, and nifedipine prevented the occurrence of AS by inhibiting pulmonary vascular remodeling [8]. Expression of hypoxia-inducible factor 1 (HIF-1), which is a protein involved in angiogenesis, vasculature remodeling, and vascular tone increase, was found to be markedly increased in the lungs and heart of broilers with AS [9,10]. Meanwhile, chemical mediators of vasoconstriction, such as endothelin-1 (ET-1), 5-hydroxytryptamine (5-HT), and endotoxin, could cause excessive resistance to blood flow by eliciting vasoconstriction and the proliferation of pulmonary smooth muscle cells (PSMCs). And those mediators been found to be overexpressed in the lungs, heart, and serum of broilers with AS [3,11–13]. Despite this, there have been few transcriptome-level studies of wholesale changes in gene expression in AS or of the relationship between the pulmonary artery and AS progression.

Evidence is mounting that the immune system also plays an important role in the etiology and progression of AS. Previous reports about the accumulation of various inflammation cells (T cells, B cells, and macrophages) in the lung from broilers with AS demonstrate this truth [3]. The differentially expressed cytokines, such as interleukin (IL)-1β, IL-8, IL-6 and chemokines like CCL5, RANTES, CXC3L1, fractalkine, monocyte chemoattractant protein (MCP)-1, and tumor necrosis factor (TNF-α) in pulmonary hypertension lesions affecting the heart, pulmonary artery and lung [12,14,15]. However, the mechanism by which immune/inflammatory response-related cytokines act through signaling pathways in broilers is not yet clear.

With the development of high-throughput sequencing technology (Illumina/Solexa), an increasing number of animal studies have adopted RNA-seq analysis to propel progress in different domains of research. However, until now, there has been just one report of liver transcriptome profiling in broilers with AS aimed at exploring the mechanisms of AS pathogenesis using genome wide expression analysis [16]. No transcriptomics study of the changes in pulmonary artery in broilers with AS has been published. Thus, in this study, we comprehensively investigated changes in the pulmonary artery of broilers with AS on the transcriptome level via high-throughput sequencing. We then integrated important pathways and significantly differentially expressed genes expressed in the pulmonary artery during the development of AS.

## Methods and Materials

### Ethics statement

All animal experiments were approved by the Institutional Animal Care and Use Committee of Jiangxi Agricultural University. All procedures and care of the chickens were carried out in strict accordance with the guidelines of the Institutional Animal Care and Use Committee of Jiangxi Agricultural University. All efforts were made to minimize the suffering of the animals.

### Animals and sample collection

Seventy-five 21-day-old Arbor Acre healthy male commercial broiler chickens with similar body weight were randomly divided into two groups, with 20 birds in the normal control group and 55 in the disease group. The birds were reared in pens (1.3×0.6×0.7m) with appropriate density. Before 21-day-old, all broilers were under room temperature 20-23C. Starting from the age of 21-day-old, the birds in the normal group were still maintained at room temperature 20-23C and were provided with tap water, while the broilers in the disease group were controlled at a low temperature (approximately 13-14C) and given the water containing 0.3% salt [10,16]. All birds had a 23 h fluorescent illumination per day throughout the trial period, and they have free access to same diet ad libitum, a special kind of pellet feed for broilers. Because the AS peak was at approximately 39-day-old in broilers [16], so we began to took samples when birds were 28-day-old and ended before 49-day-old. For timely founding AS broilers and getting samples, before feeding the birds (three times a day), we carefully observed whether broilers displayed symptoms such as AS-like depression, reluctance to move, open-beak breathing, distended abdomen, or cyanosis. Once these symptoms were observed e birds were immediately weighed and 2 mL of blood per broiler was taken from the brachial vein and collected in a tube coated with heparin for a routine blood examination by Auto Hematology Analyzer (PE-6300 VET, China). Blood parameter data was analyzed by using SPSS version 17.0 (SPSS Inc., Chicago, IL, USA), with a P value less than 0.05 being considered statistically significant different while P<0.01 being considered highly significantly different. After blood sample collection, the broilers were sacrificed by jugular bleeding and the pulmonary artery was collected immediately and quickly rinsed in saline water. The artery was then cut into 4 pieces, placed in tubes, and immersed in liquid nitrogen for 10 minutes before being placed in a -80C freezer until RNA sequencing. Meanwhile, a piece of pulmonary artery tissue was cut and stored in 10% formaldehyde for pathological observation. To calculate the Ascites Heart index (AHI), after removing the atrium and aorta, the right ventricle (RV) was separated from the septum and left ventricle, then the RV (right ventricle) and TV (total ventricle) weights were determined and the ratio of RV:TV was calculated. The normal AHI was less than 0.219, while AS birds had an AHI greater than 0.249.

Based on the pathological changes observed (i.e., yellow liquid pooling in the abdominal cavity and pericardium), the analysis of AHI (over 0.249) and the routine blood testing data (high hematocrit and hemoglobin levels), finally two pulmonary arteries samples from two AS-positive birds with the AHI of 0.380 and 0.301 separately, and other two pulmonary artery samples from two normal birds were selected for histopathological examination and RNA-seq testing.

### RNA preparation

Total RNA was extracted from the pulmonary artery using TRIzol reagent (Invitrogen, Burlington, ON, Canada) according to the manufacturer’s protocol. RNA contamination and degradation were monitored on 1% agarose gels. We used a Nano Photometer® spectrophotometer (IMPLEN, CA, USA) to determine the RNA purity. RNA concentration and integrity were separately measured by a Qubit® RNA Assay Kit on a Qubit® 2.0 Fluorometer (Life Technologies, CA, USA) and an RNA Nano 6000 Assay Kit on the Bioanalyzer 2100 system (Agilent Technologies, CA, USA) according to the manufacturers’ recommendations.

### Library preparation and RNA-sequencing

A total of 3 μg RNA per sample was used as input for RNA sample preparation. Sequencing libraries were generated using the NEBNext® Ultra™ RNA Library Prep Kit for Illumina® (NEB, USA) following the manufacturer’s recommendations, and index codes were added to attribute the sequences to each sample. In brief, mRNA was purified from total RNA using poly-T oligo-attached magnetic beads, and fragmentation was performed using divalent cations at an elevated temperature in NEBNext First Strand Synthesis Reaction Buffer (5X). PCR was performed using Phusion High-Fidelity DNA polymerase, Universal PCR primers and an Index (X) Primer. PCR products were purified (AMPure XP system) and library quality was assessed on the Agilent Bioanalyzer 2100 system. The clustering of the index-coded samples was performed on a cBot Cluster Generation System using a TruSeq PE Cluster Kit v3-cBot-HS (Illumina) according to the manufacturer’s instructions. After cluster generation, the library preparations were sequenced on an Illumina HiSeq 2500 platform at the Novogene Company, Beijing, China, and 50 bp single-end reads were generated.

### Data Analysis

#### Quality control and read mapping to the reference genome

Raw data of FASTQ format in the current trial has been deposited in Short Read Archive (SRA) database of the National Center for Biotechnology Information (NCBI) with an accession number SRP068247. Raw data were firstly processed through in-house scripts, and clean reads were obtained by removing those containing adapters or poly-N sequence as well as those of low quality from the raw data in this step. At the same time, Q20, Q30 and GC content of the clean data were calculated. All the downstream analyses were based on the clean data with high quality. Reference genome and gene model annotation files were downloaded from the genome website directly (ftp://ftp.ensembl.org/pub/release-76/fasta/gallus_gallus/dna/). Indexing of the reference genome was performed using Bowtie v2.0.6, and 50 bp single-end clean reads were aligned to the reference genome using TopHat v2.0.9 with the mismatch being set as 2. We selected TopHat as the mapping tool because it can generate a database of splice junctions based on the gene model annotation file, which results in a better mapping result than those obtained with other non-splice mapping tools.

#### Quantification of gene expression level and identifying differentially expressed genes

HTSeq v0.6.1 was used to count the reads member mapped to each gene. And then the RPKM (Reads Per Kilobase of exon momdel per Million mapped reads) of each gene was calculated based on the length of the gene and the read count mapped to this gene. RPKM, was used to consider the effect of sequencing depth and gene length at the same time, a commonly used method for estimating gene expression levels [17]. Genes with high RPKM values means that these genes express at a high level.

Differential expression analysis of two biological replicates per condition was performed by using the DESeq R package (1.10.1) [18]. DESeq provides statistical routines for determining differential expression in digital gene expression data using a model based on the negative binomial distribution. The resulting P-values were adjusted using Benjamini and Hochberg’s approach for controlling the false discovery rate. Genes with an adjusted P-value < 0.05 (Padj) were considered differentially expressed.

#### GO and KEGG enrichment analysis of differentially expressed genes

Gene Ontology (GO) enrichment analysis of differentially expressed genes was implemented by the GOseq R package [19], in which gene length bias was corrected. GO terms with a corrected P value < 0.05 were considered significantly enriched for the differentially expressed genes. KEGG (Kyoto Encyclopedia of Genes and Genomes) is a database resource for understanding high-level functions and utilities of biological systems (http://www.genome.jp/kegg/).) KOBAS software (KOBAS, Surrey, UK) was used to test for statistically significant enrichment of differentially expressed genes in KEGG pathways.

#### Validation of RNA-seq data by real-time quantitative PCR (qRT-PCR)

To validate the accuracy of RNA-seq, we chose 12 genes distributed at 4 RPKM intervals (1–3, 3–15, 15–50, and > 50) for qRT-PCR. The samples were same as the ones for the RNA-seq analysis. The primer pairs for the selected genes were designed using Primer 5 and are shown in S1 Table. The cDNA was synthesized from 1 μg of total RNA using the PrimerScript RT Reagent Kit (Takara, China). The amount of amplified DNA was monitored by fluorescence at the end of each cycle using a 7500 Real-Time PCR system. Each group consisted of two samples with three replicates each. The expression level of each gene was evaluated by the 2^-ΔΔCt^ method.

### Histopathological observation of the pulmonary artery

To evaluate the histopathological changes in the pulmonary arteries of broilers with AS, pulmonary arteries stored in 10% formaldehyde were transferred to 4% Paraformaldehyde solution for more than 24 hours to fully fix the structure, protein and cell morphology of the tissue. Then, the tissues were placed in an ascending gradient of ethanol (70%-99.5%) for dehydration and were made transparent by dipping in xylene three times (for 4 minutes, 2 minutes, and 30 seconds). Next, they were placed in two beakers filled with paraffin for 1 hour, routinely sectioned and stained using hematoxylin eosin. The stained sections were observed by microscopy at 200X magnification to examine changes in the tissue, and the sections were photographed.

## Results

### AHI and blood routine parameters indicating AS

During the experiment, two broilers in the disease group died, possibly from AS; after necropsy, both showed fiber exudation in the abdominal cavity and pericardium, and their AHIs were over 0.249. The average weight of chickens in disease group was 2.004kg and normal group was 2.217kg. In Table 1, it can be seen that the AHI, hemoglobin (HGB) and hematocrit (HCT) values indicating AS were extremely higher in the disease group compared to the control group during the whole process from day 21 to day 49 (P<0.01). In addition, some parameters related to immune/inflammation, such as white blood cell (WBC), lymphocyte (LYM), and Granulocyte ratio (GRNA), were also extremely and significantly increased (P<0.01), and the red blood cell distribution width-standard deviation (RDW-SD) significantly increased (P<0.05) when compared with the normal birds.

**Table 1:** Summary of AHI and blood routine in disease and normal groups.

Project name	Normal group (n = 20)	Disease group (n = 55)	P-value
**RV/TV (g/g)**	0.21±0.01	0.27±0.01	<0.01
**HGB (g/L)**	151.00±5.01	200.45±6.33	<0.01
**HCT (%)**	29.75±1.09	35.47±1.02	<0.01
**WBC (10**^**9**^**/L)**	104.96±5.59	180.50±12.32	<0.01
**LYM (10**^**9**^**/L)**	94.59±4.46	140.67±6.76	<0.01
**GRNA (%)**	7.04±0.77	22.16±2.94	<0.01
**RDW-SD (fL)**	30.47±0.41	32.50±0.67	<0.05

**Note**: “n” represents the number of broilers in each group. RV/TV means right ventricular weight/ total ventricular weight; HGB means hemoglobin; HCT means hematocrit; WBC means white blood cell; LYM means lymphocyte; GRNA means Granulocyte ratio; RDW-SD means red blood cell distribution width-standard deviation.

### RNA-seq results

#### Transcriptome sequencing and read mapping to the reference genome

An overview of the sequencing and mapping results is provided in Table 2. As shown, after filtering out the adaptor, poly-N sequences and low-quality reads, 12,921,832 (N1), 15,674,675 (N2), 13,493,794 (D1), and 13,735,361 (D2) clean reads were retained. The GC content, Q20 and error rate reflecting the quality of the libraries and sequence data were determined. Almost all clean reads (90.45% of N1, 90.35% of N2, 89.82% of D1, and 89.66% of D2) mapped to the reference genome. According to the requests for the samples, our reads can be used to take the analysis further. The Pearson correlation coefficient for the replicates indicated that our sequencing data were reliable (S1 Fig).

**Table 2:** Basic summary of sequence and sequencing reads mapping to reference genome.

Sample name	N1	N2	D1	D2
**Raw reads**	13172095	15908860	13818353	13992954
**Clean reads**	12921832	15674675	13493794	13735361
**Clean bases**	0.65G	0.78G	0.67G	0.69G
**GC content (%)**	48.61	49.01	50.26	50.42
**Multiple mapped**	218094(1.69%)	228240(1.46%)	210695(1.56%)	250856(1.83%)
**Uniquely mapped**	11469582(88.75%)	13933328(88.89%)	11909334(88.26%)	12064522(87.84%)
**Reads map to ‘+’**	5734482(44.38%)	6963551(44.43%)	5955487(44.14%)	6029451(43.9%)
**Reads map to ‘-’**	5735100(44.38%)	6969777(44.47%)	5953847(44.12%)	6035071(43.94%)
**Non-splice reads**	9790044(75.76%)	11774512(75.12%)	10000081(74.11%)	10074221(73.35%)
**Splice reads**	1679538(13%)	2158816(13.77%)	1909253(14.15%)	1990301(14.49%)

**Note**: “+” refers to sense strands, “-” indicates anti-sense strands; “non-splice reads” refers to entire sequence is mapped to one exon; “splice reads” refers to reads mapped to the border of exon. “N1, N2” represents normal samples and “D1, D2” represents disease samples.

#### Differential expression analysis

**Fig 1:**
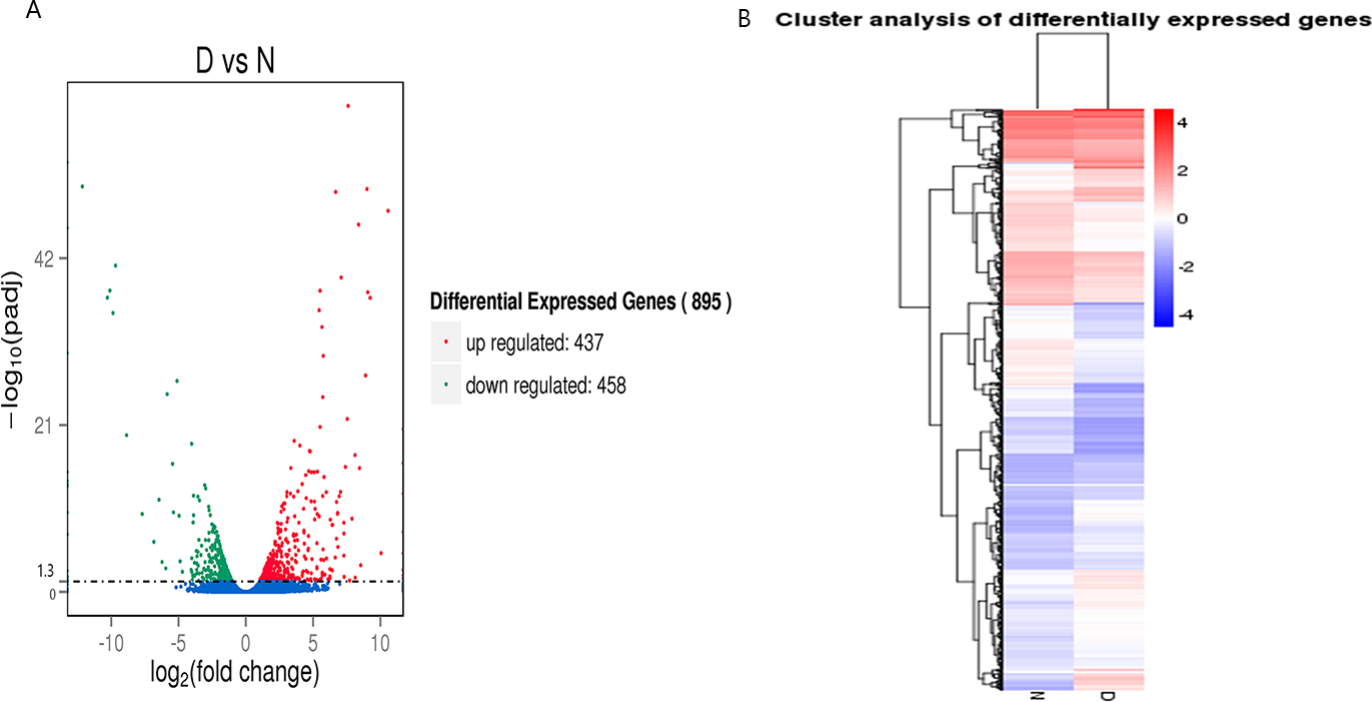
Differential expressed genes filtering. **(A)** In the volcano diagram, the significantly up-regulated genes are in red and the down-regulated genes are in green, while no difference is indicated in blue by the standard of p-adjusted< 0.05. **(B)** A heat map of N and D sample gene expression clustering, with high levels of expression shown in red and low levels shown in blue.

Eight hundred ninety-five genes that were differentially expressed in the pulmonary arteries between diseased and normal broilers were identified, with 437 up-regulated and 458 down-regulated genes by a threshold of padj < 0.05 (Fig 1A). The gene expression density distributions between disease and normal samples are shown (S2 Fig). To understand the expression patterns of the differentially expressed genes, we performed a hierarchical clustering analysis of those differentially expressed genes based on the RPKM values for the genes in the four samples (Fig 1B). Both disease and normal samples had high and low expressed genes, implying that the development of AS relies on various mechanisms.

#### Functional annotation and pathway assignment of DEGs and key DEGs

**Fig 2:**
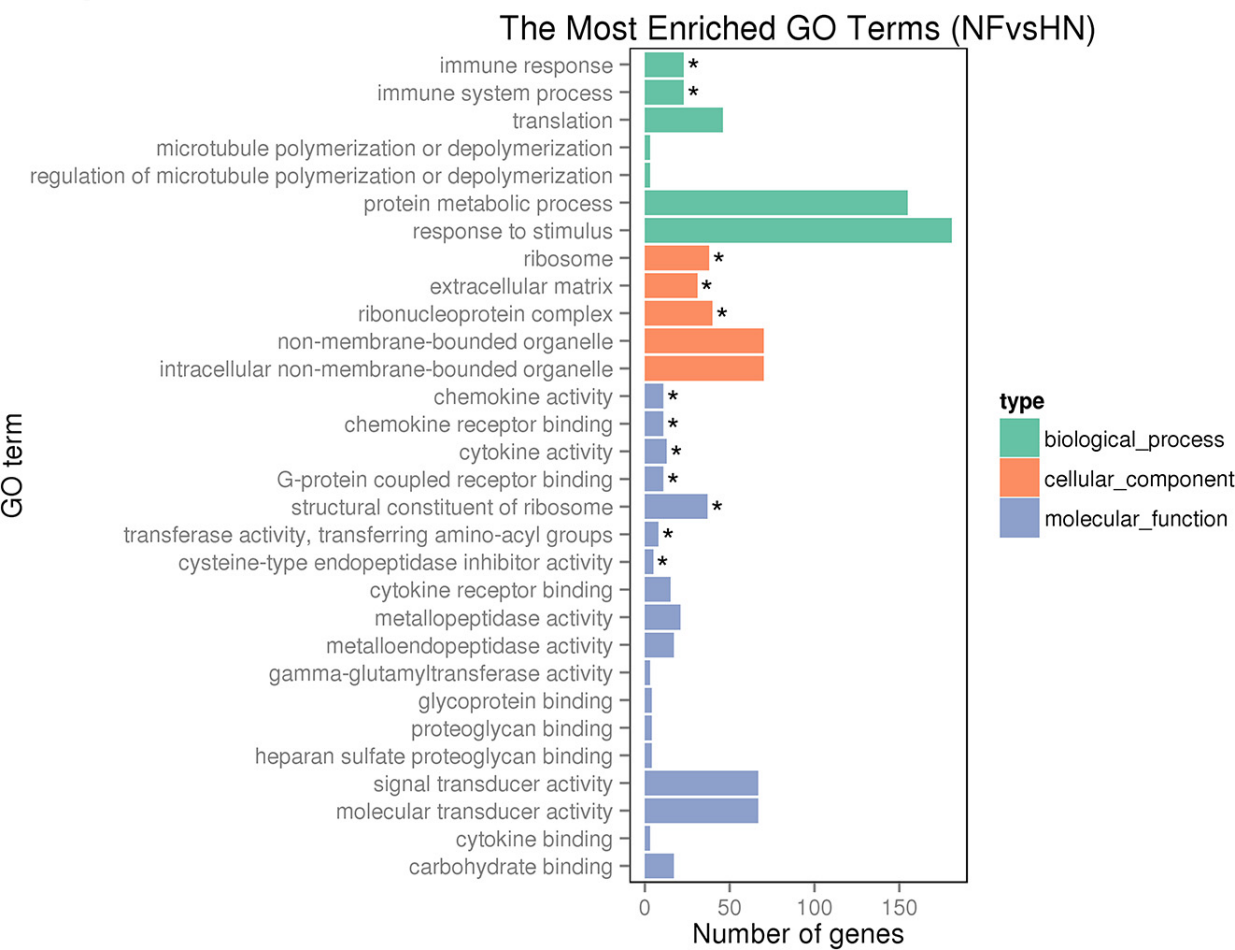
GO enrichment analysis of DEGs in the pulmonary artery. The three main categories used for GO analysis. The y-axis and x-axis indicate subcategory name and gene number, respectively. * represents the significantly enriched GO terms.

To illustrate the functional associations of the 895 significantly differentially expressed genes (DEGs), the DEGs were subjected to GO analysis and were found to fall into three main categories: biological process, cellular component, and molecular function (Fig 2). Six significantly enriched GO terms in the biological process and molecular function categories were related to immune function, including immune response and immune system process, chemokine activity, chemokine receptor binding cytokine activity and cytokine receptor binding. And 13 DEGs related to cytokines were repeatedly enriched to those six GO terms and they were shown in S3 Table. And there are three significantly enriched GO terms in the cellular component category, the ribosome, extracellular matrix, and ribonucleoprotein complex, which were related to pulmonary artery remodeling and proliferation. Notably, the significantly enriched GO terms associated with the immune system were up-regulated, while cell structure-related GO terms were more often associated with down-regulated genes (S3 Fig).

**Fig 3:**
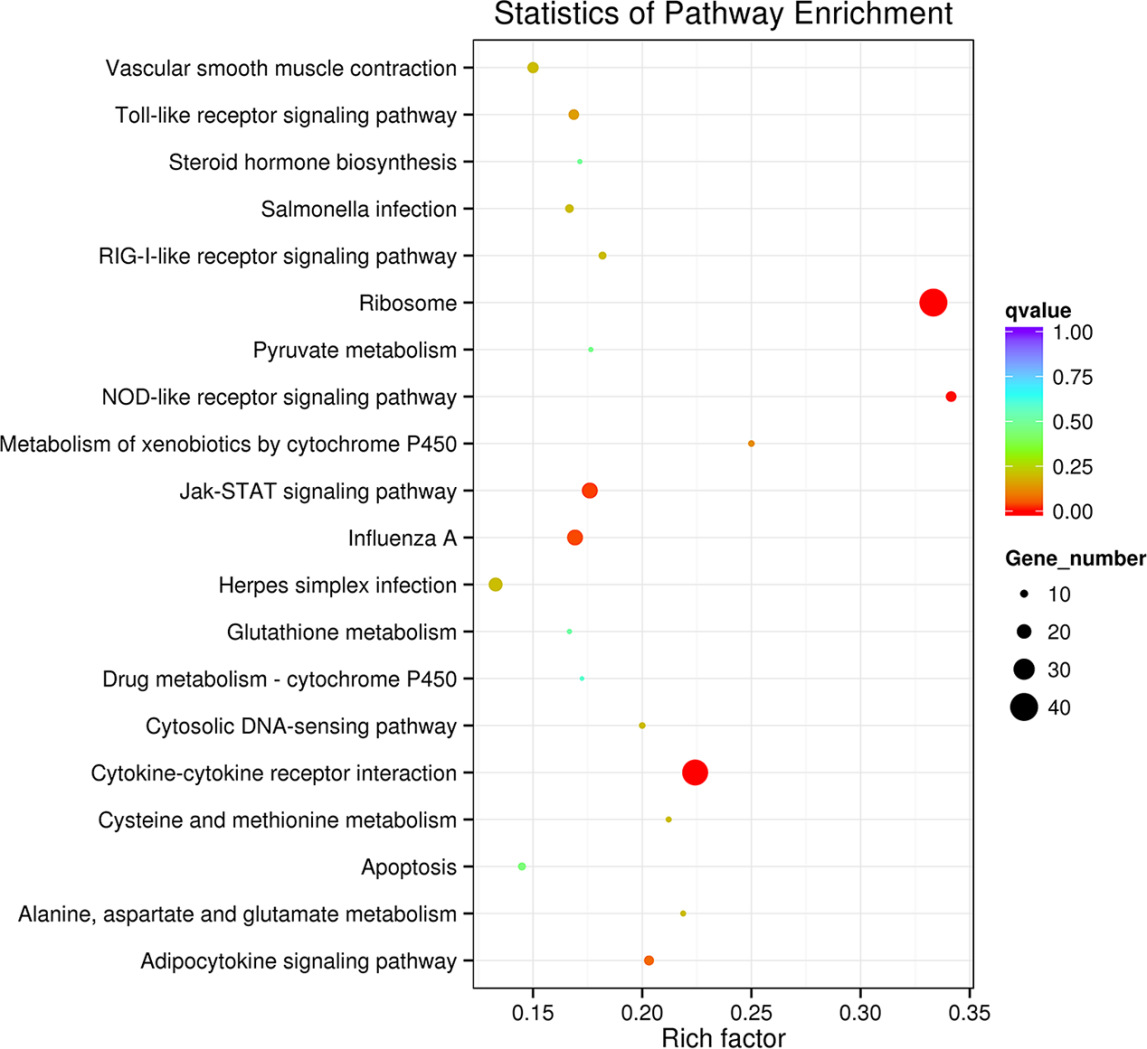
KEGG enrichment analysis of DEGs in the pulmonary artery. The most 20 KEGG pathways were presented. The y-axis and x-axis indicate pathway name and rich factor, respectively. The size of circle dot means gene number.

The KEGG analysis revealed that those 895 DEGs were mapped to 121 canonical reference pathways and the most top 20 enriched key pathways were presented in the S4 Table and Fig 3. The dominant pathway was ribosome with 40 down-regulated genes (S5 Table), and 35 genes of them were also enriched to the GO term-ribosome, indicating that the ribosome may be involved in the pulmonary artery remodeling in AS progression. The Jak-STAT (Janus kinase-signal transducer and activator of transcription) signaling pathway (S4 Fig), a major mechanism used to transmit signals from extracellular receptors to the nucleus and regulate some major biological processes including pulmonary artery remodeling and proliferation, immune response, was also extremely enriched with 22 DEGs (Table 3). And it includes some key genes which significantly changed, like the upstream up-regulated IL-6, 13 downstream cytokine receptor-related genes, STAT3, STAT5, SOCS 3 and Pim-1. Other two highly enriched pathways were cytokine-cytokine receptor interaction and NOD-like receptor signaling pathway which was highly related to pulmonary artery remodeling, with 35 up-regulated genes (S6 Table) and 13 up-regulated genes (Table 3, S5 Fig), respectively. The enriched NOD-like receptor signaling pathway showed three overexpressed components: (1) IKKα and IB; (2) P38; and (3) five cytokines IL-6, IL8, IL-1β, IL-18, and MIP-1β. Typically, 13 DEGs enriched to 6 highly enriched GO terms, 13 cytokine receptors genes involved to Jak-STAT signaling pathway, and five cytokines enriched in NOD-like receptor signaling pathway all enriched to the cytokine-cytokine receptor interaction (S6 Table). This annotations of DEGs provide a valuable resources for further investigating specific process, pathways that were involved in the AS progression.

**Table 3:** Putative significantly differential expressed molecules and genes enriched to Jak-STAT signaling pathway and NOD-like signaling pathway.

Signaling molecules	Gene name	Gene ID	Padj	Description
**Jak-STAT signaling pathway**				
IL6	CSF3	ENSGALG00000026420	0.00010085	Interleukin-6/Interleukin-23
	IL6	ENSGALG00000010915	2.23E-06	Interleukin-6/Interleukin-23
CytokineR	IL4RA	ENSGALG00000006313	0.02562	Interleukin-4 receptor alpha
	IL2RG	ENSGALG00000005638	0.0013193	Interleukin-6 receptor alpha
	IL13RA1	ENSGALG00000006032	0.022197	Interleukin-6 receptor alpha
	IL13RA2	ENSGALG00000020316	1.74E-21	Interleukin-6 receptor alpha
	IL12RB1	ENSGALG00000027301	0.028163	Fibronectin, type III
	IL20RA	ENSGALG00000013869	4.37E-13	Interferon alpha/beta receptor
	IFNGR1	ENSGALG00000013865	0.012232	Interferon gamma receptor
	IL10R1	ENSGALG00000024075	0.00027589	Interferon alpha/beta receptor
	GP130	ENSGALG00000014716	0.013903	Immunoglobulin C2-set-like
	IL22RA2	ENSGALG00000013868	0.038518	Interferon alpha/beta receptor
	CSF3R	ENSGALG00000002112	1.54E-10	Immunoglobulin C2-set-like,
	OSMR	ENSGALG00000003747	0.032166	Fibronectin, type III
	IL10R2	ENSGALG00000015941	0.024509	Interferon alpha/beta receptor, beta
STAT	STAT5	ENSGALG00000003282	0.0077515	STAT transcription factor
	STAT3	ENSGALG00000003267	0.00026403	STAT transcription factor
PI3K	PIK3R5	ENSGALG00000021573	2.22E-05	Phosphoinositide 3-kinase regulatory subunit 5/6
SOCS	SOCS3	ENSGALG00000027786	4.37E-12	SOCS protein,
	SOCS1	ENSGALG00000007158	1.38E-08	SOCS protein
Pim-1	PIM1	ENSGALG00000000742	3.42E-05	Lipopolysaccharide kinase
CycD	CCND3	ENSGALG00000003485	0.00056472	Cyclin A/B/D/E/F
**NOD-like signaling pathway**				
IKKγ	CHUK	ENSGALG00000003289	0.0019618	Serine/threonine/dual specificity protein kinase
P38	MAPK11	ENSGALG00000008612	0.0003684	Mitogen-activated protein (MAP) kinase, p38
IγK	NFKBIA	ENSGALG00000027864	0.046652	Ankyrin repeat-containing domain
IL8	IL8	ENSGALG00000026098	5.93E-47	Chemokine interleukin-8-like domain
	K60	ENSGALG00000011668	3.31E-21	Chemokine interleukin-8-like domain
IL6	IL6	ENSGALG00000010915	2.23E-06	Interleukin-6/Interleukin-23
IL1β	IL-1BETA	ENSGALG00000000534	1.86E-18	Interleukin-1 alpha/beta
IL18	IL18	ENSGALG00000007874	0.020274	Interleukin-18 Interleukin-1 family
NOD1	NOD1	ENSGALG00000011535	0.0077515	P-loop containing nucleoside triphosphate hydrolase
PIP2	RIPK2	ENSGALG00000015899	0.00045037	Serine/threonine/dual specificity protein kinase
cIAP12	ITA	ENSGALG00000017186	2.53E-09	Baculoviral inhibition of apoptosis protein repeat
A20	TNFAIP3	ENSGALG00000013861	5.22E-10	Ovarian tumour, otubain
Cardinal	CARD8	ENSGALG00000007698	0.048385	Death-like /CARD domain
HSP90	HSP90AA1	ENSGALG00000011351	0.049445	Heat shock protein Hsp90 family

**Note:** A gene with a Padj<0.05 is considered as significantly differential expressed. Padj means the corrected-P value.

Besides those DEGs that were significantly enriched to some key functional groups, we also identified other 7 specific DEGs (Table 4) that regulated pulmonary artery remodeling and vascular contraction in this study. In this study, except for those significantly enriched KEGG pathways, we also noticed other important functional groups with specific DEGs, like immune response related pathways (Toll/RIG -like receptor and MAPK signaling pathways) in the S7 Table; fatty acid and ammo acid metabolism related pathways (peroxisome, adipocytokine, arginine and proline synthesis, tryptophan synthesis et.al) in the S8 Table; pulmonary artery contraction related pathways (vascular smooth muscle contraction, calcium signaling pathway, and transforming growth factor β (TGF-β) signaling pathway et.al) in S9 Table.

**Table 4:** Other putative significantly differential expressed genes regulating pulmonary artery remodeling and vascular contraction in this study.

Gene name	Gene ID	Readcount D	Readcount N	Description	Padj
CYP1B1	ENSGALG00000025822	398.6948	1071.227	Cytochrome P450, E-class, group ICytochrome P450, E-class, group IV	0.003082
ALDH7A1	ENSGALG00000008229	1946.312	856.2167	Aldehyde/histidinol dehydrogenase||Aldehyde dehydrogenase domain	0.01793
MYLK	ENSGALG00000011708	13269.39	40167.41	Immunoglobulin Serine/threonine/dual specificity protein kinase, catalytic domain	8.22E-05
CaM	ENSGALG00000010023	2193.544	4622.791	EF-hand, Ca insensitive||EF-hand domain	0.028664
CAMK4	ENSGALG00000000244	12.22596	63.68015	Protein kinase-like domain|| Serine /threonin dual specificity protein kinase	0.026825
INOS	ENSGALG00000005693	8249.399	188.7036	Nitric oxide synthase, N-terminal|| Nitric-oxide synthase, eukaryote	3.54E-36
BMP7	ENSGALG00000007668	219.6748	591.5117	Cystine-knot cytokine||Transforming growth factor-beta, C-terminal/N-terminal	0.00706

**Note:** A gene with a Padj<0.05 is considered as significantly differential expressed. Padj means the corrected-P value. Readcount D means the readcounts of disease samples; Readcount N means the readcounts of normal samples.

### Validation of RNA-seq data by real-time quantitative PCR (qRT-PCR)

**Fig 4:**
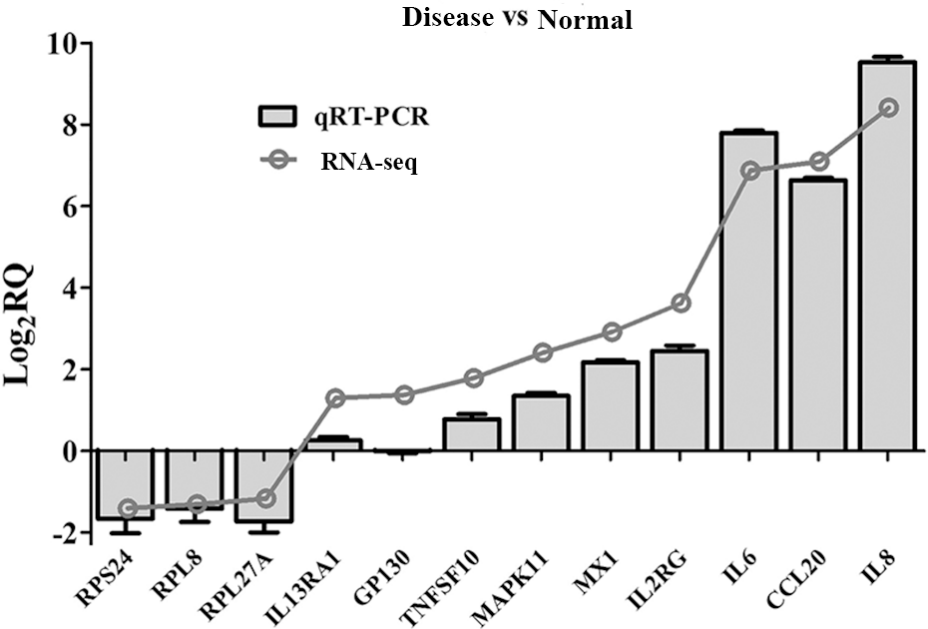
qRT-PCR and sequencing. qRT-PCR validation of differentially expressed genes in disease and normal samples of pulmonary artery for 12 genes.

To validate the accuracy of the RNA-seq data, we chose 12 genes for qRT-PCR analysis. As presented in Fig 4, only GP130 and IL13RA1 showed different expression levels between qRT-PCR and RNA-seq detection, but the general trends of up and down-regulation of those selected genes were consistent. Thus, qRT-PCR verified the reliability and accuracy of our transcriptome profiling.

### Pathological observation of the pulmonary artery

**Fig 5:**
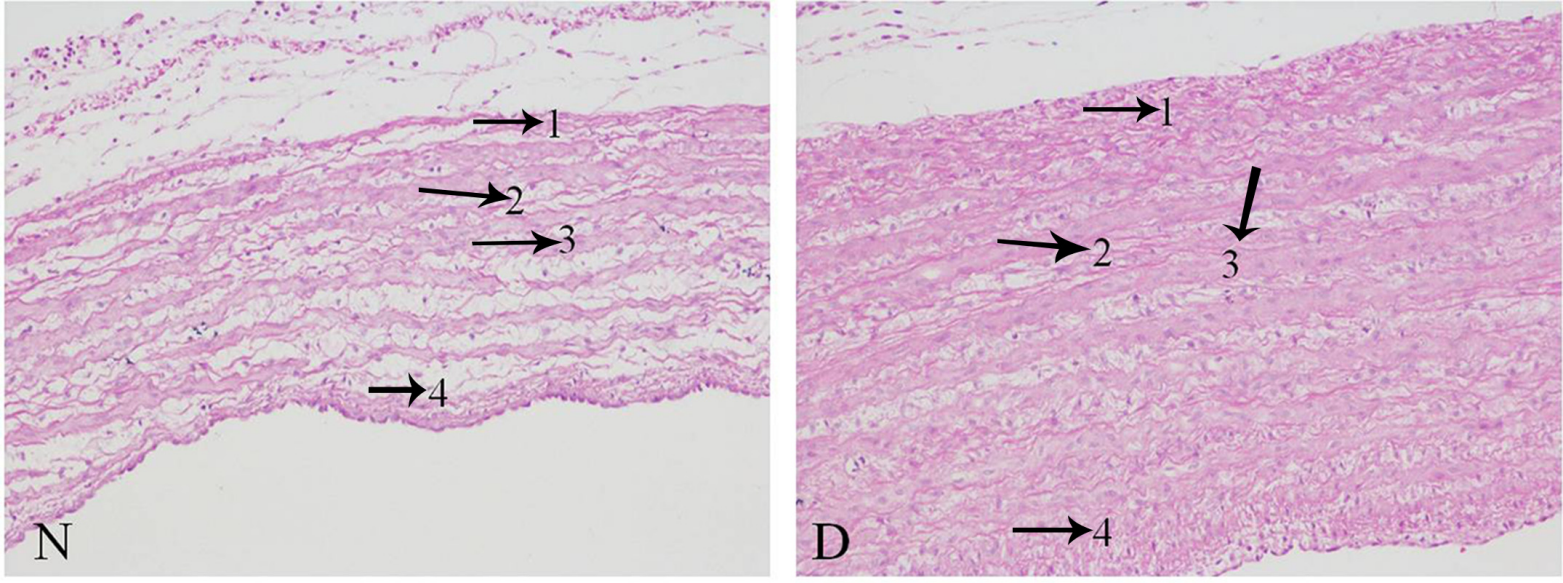
**Observation of pulmonary artery sections** (200X, HE) The pulmonary artery wall thickness of disease (D) is noticeably increased. In the D sample, 1) the tunica adventicia was more compact and exhibited increased connective tissue; 2) the smooth muscle fiber was thicker; 3) there was excessive fiber production; and 4) the intima was more compact. The arrows indicate the pathological changes.

From Fig 5, showing pulmonary artery changes on the pathological level, it was noticed that the thickness of the pulmonary artery in broilers with AS was markedly elevated, and marginal smooth muscle layers were discontinuous and disordered. Furthermore, the mesenchyme between the muscle layers was more loosely associated in the broilers with AS than in normal birds.

## Discussion

In the present study, an AS model was successfully established and pulmonary arteries from AS broilers were obtained to investigate the relation between the pulmonary artery remodeling and AS on the transcriptome level using high-throughput sequencing technology. To correctly identify AS broilers prior to collecting samples for RNA-seq, we utilized common parameters such as yellow liquid accumulation in the abdominal cavity and pericardium, AHI (RV/TV)>0.249, and increased HCT and HGB levels when compared to normal birds [4,20,21]. These parameters supported our present results regarding RV/TV and the difference in routine blood indexes, such as HCT and HGB, between disease and control groups (Table 1). Through RNA-seq analysis in AS and normal broilers’ pulmonary arteries, we observed that a total of 895 significantly differentially expressed genes, which are highly enriched to 12 GO terms and 4 KEGG pathways (Padj<0.05) regulating pulmonary artery remodeling and consequently occurrence of AS. These GO terms and pathways include ribosome, Jak-STAT and NOD-like receptor signaling pathways which regulate pulmonary artery remodeling through vascular smooth cell proliferation, inflammation and vascular smooth cell proliferation together.

### Ribosome

The ribosome has been reported to be associated with cell growth, proliferation, and apoptosis [22]. Importantly, ribosome pathway factors were recently reported to mediate the formation of the carotid intima in mice in response to injury, and ribosomal protein L17 (RPL17), similar to tumor suppressors, can inhibit VSMC cell cycle progression [23]. In addition, decreased expression of RPL17 leads to VSMC proliferation [23]. Based on the current GO classification (Fig 2, S3 Fig) and KEGG analysis (Fig 3), 40 down-regulated genes including RPL 5, 7, 8, 9, 14, 21 were significantly enriched in the ribosome functional group (S5 Table), indicating that the ribosome may be associated with pulmonary artery remodeling through vascular smooth muscle cell (VSMC) proliferation. Indeed, the present pathological study demonstrated that the pulmonary artery was remodeled via vascular smooth muscle cell proliferation. Pulmonary vascular remodeling was confirmed as an important pathological contributor to AS that leads to an increase in pulmonary vascular resistance, prolonging elevated arterial hypertension [24]. The relationship between ribosomal protein function and VSMCs has not been well studied until now, so our results concerning ribosomal proteins are very valuable for further investigating vascular biology and providing a potential therapeutic target to prevent PH on both humans and broilers.

### JAK-STAT signaling pathway

In the present study, with regard to the significantly enriched Jak-STAT signaling pathway (S4 Fig), a series of up-regulated genes were noticed, including IL-6, CSF3, 13 cytokine receptor-related genes, STAT3, STAT5 and final Pim1, SOCS3 and SOCS1 (Table 3). It was already reported that the expansion of upstream cytokine receptors is pivotal for the variable functions of Jak-STAT signaling pathway [25]. Jak-STAT signaling activation depends on the binding of extracellular cytokines to cytokine receptors on the cell membrane, leading to intracellular JAK phosphorylation and activation, finally causing STAT to be phosphorylated and two activated STATs to bind to one another and translocate to the nucleus, where they interact with accessory factors to promote the transcription of target genes [26]. Activated NOD-like receptor signaling pathway was found to promote the expression of various cytokines, such as IL-6, in the present study. Actually, 13 upregulation of cytokine receptors genes (IL4RA, IL2RG, IL13RA1, IL13RA2, IL12RB1, IL20RA, IFNGR1, IL10R1, GP130, IL22RA2, CSF3R, OSMR, IL10R2) were enriched in the Jak-STAT signaling pathway, and they were also found in the Cytokine-cytokine receptor pathway (S6 Table). And the activated Jak-STAT signaling pathway has a direct impact on gene transcription and activates the expression of a wide range of transcription factors and proteins, such as HIF-1α, nuclear factor of activated T cells (NFAT), Pim1, and Krueppel-like factor 5 (KLF5), thereby mediating cellular activities such as proliferation, differentiation and survival and regulating immune/inflammatory responses and other pathological processes[26–29]. All of these cellular activities are key contributors to pulmonary artery remodeling underlying AS progression [30]. And the up-regulated Pim1 was also found in this study. At the same time, the activated Jak-STAT pathway can be extinguished through negative regulation by inhibitors, including latent SHPs or SOCSs, via recruitment of the receptor signaling complex [26]. This last point is supported by our results showing that SOCS3 and SOCS1 were up-regulated in pulmonary artery from AS broilers, indicating the important role of SOCSs in pulmonary artery remodeling through its regulation of the JAK-STAT signaling pathway.

### NOD-like receptor signaling pathway

Different Pattern Recognition Receptors (PRRs) including NOD-like receptor recognize different types of pathogen targets and then activate the downstream protein kinases and transcription factors such as NF-κB, finally triggering the increased expression and release of cytokines and activating the related immune defense system[31]. In both broilers and humans, pulmonary artery hypertension has been shown to involve a critical immune system/inflammatory component and immunopathology [3]. And the pulmonary artery remodeling, which also serves as a key step to the progression of pulmonary hypertension in broilers, was reported to result from smooth muscular cell proliferation and inflammation cells infiltration [30]. It can be suggested that the immune response/inflammatory involved in the development of AS by promoting pulmonary artery remodeling. In the present KEGG analysis, the significantly enriched NOD-like receptor signaling pathway expressed three overexpressed elements (Table 3, S5 Fig): (1) IKKα and IκB; (2) P38; and (3) cytokines, IL-6, IL8, IL-1β, IL-18, MIP-1β. NOD-like receptors have been indeed detected in avian species [32]. When responding to extracellular stimuli such as inflammatory cytokines, the activated IKK induces the phosphorylation, ubiquitination and degradation of IκB, releasing NF-κB to activate immune-related gene transcription not limited to IL-1β, IL-6, IL-8, TNFα, IFNγ, MCP-1, and iNOS [33,34]. However, in the present study, both IKKα and IκB were up-regulated, but NF-κB did not alter in NOD -like receptor signaling pathway. This may because some IκB degradation does not require IKK phosphorylation or ubiquitination [33], so IκB was not down-regulated by up-regulated IKK in this model. It may also be because IκB was not the only inhibitor of NF-κB activity, so NF-κB was not down-regulated. In support of the latter explanation, recent studies have found that besides IκB, additional pleiotropic NF-κB cofactors and more complex regulators of gene expression together suppress NF-κB activity [35]. P38 proteins, a class of mitogen-activated protein kinases (MAPKs), play a key role in inflammatory/immune responses and are involved in essential cellular processes [36]. However, in the present study, unlike NF-κB, the P38 MAPKs were up-regulated, especially MAPK11, indicating that the P38 MAPKs may be more crucial to the immune response occurring in pulmonary artery remodeling in AS progression. It was reported that P38 MAPKs are activated and play a pathogenic role in vascular diseases such as PH through their involvement in vascular cell proliferation and the inflammatory response via IL-6 [37]. Furthermore, pulmonary artery vascular proliferation, vascular remodeling and inflammation have already been demonstrated to contribute to AS progression [3,24]. Consistently, both P38 and its downstream gene IL-6 were up-regulated in NOD-like receptor signaling pathways in this study. However, in addition to IL-6, we also detected other downstream overexpressed cytokines, such as IL8, IL-1β, IL-18, and MIP-1β. Actually, during the pathogenesis of inflammatory disease, P38 has been implicated in the production of various inflammatory cytokines including IL-1β, IL-18, IL-6 [38,39]. Furthermore, previous studies have reported that IL-1β could up-regulate the expression of many inflammatory chemokines, including CCL4 (MIP-1β) and CXCL8 (IL-8) in human articular chondrocytes by transcriptional regulation [40]. CCL4 produced by many cells, including fibroblasts and endothelial and immune cells, could initiate the migration of immune cells and has been reported to cause pathological changes in the pulmonary arteries of cirrhotic mice. This effect is similar to that of PH [41,42]. IL-8, IL-6, IL-1β, and IL-18 were all elevated in human and animal models with PH, and they consequently emerged as crucial contributing cytokines in the pathogenesis of PH [43]. Consistently, the existing routine blood test also demonstrated that inflammatory parameters in the AS group were significantly higher than in the control group (Table 1). Our findings provide a clear understanding of the altered immune/inflammatory mechanism involving multiple signaling pathways that function in the pulmonary artery remodeling in AS, potentially supporting new therapeutic insight into the signaling pathways and identifying key target genes.

### AS-specific gene transcripts in Pulmonary arteries

In the current study, besides those DEGs that significantly enriched to GO terms and KEGG pathways, we also observed other important DEGs (CYP1B1, ALDH7A1, MYLK, CaM, CAMK4, iNOS and BMP7) that play a key role in the pulmonary artery remodeling vascular contraction. Both contribute to the occurrence of AS by elevating vascular resistance. CYP1B1 was down-regulated and ALDH7A1 was up-regulated in the pulmonary artery tissues from AS broilers. It was reported that a lack of CYP1B1 expression in smooth muscle cells lead to vascular dysfunction and a loss of vascular integrity via the regulation of the proliferation, migration, and survival of smooth muscle cells in mice[44]. This report supports our finding that CYP1B1 is down-regulated in pulmonary arteries during AS progression. ALDH7A1 knockdown has been shown to cause reduced cell proliferation in the optic cup of zebrafish [45] and functions in lipid peroxidation in chickens [46]; cell proliferation are closely linked to pulmonary artery remodeling. Thus, this suggests that ALDH7A1 can serve as a promising gene target for preventing AS progression. MYLK, CaM and CAMK4 thsee three genes were all down regulated in this study. In human pulmonary artery smooth muscle cells, when the concentration of cytoplasmic Ca^2+^ increases, Ca^2+^ ions bind to calmodulin (CaM) and activate downstream myosin light chain kinase (MYLK), thereby causing the phosphorylation of the myosin light chain and finally resulting in the sliding motion of the actomyosin complex that leads to pulmonary vasoconstriction [47]. In addition, Ca^2+^ also activates intracellular Ca^2+^-dependent signal transduction proteins such as CaM kinase (CaMK) and MAPK, which trigger PAMSC entry into the cell cycle from a quiescent differentiated state, thereby leading to proliferation [47]. The calcium signaling pathway is closely associated with pulmonary vascular smooth muscle contraction and remodeling, both of which are characteristics of AS [47]. This is because the calcium signaling pathway is involved in the development of AS in broilers via Ca2+ influx that leads to increased Ca2+ levels in the cytoplasm, which in turn stimulates pulmonary artery smooth muscle cell contraction, further elevating the vascular resistance to blood flow and pulmonary hypertension [48]. iNOS has been demonstrated to be closely related to AS progression, because its product, nitric oxide (NO), could induce vasodilation and attenuate pulmonary arteriole remodeling [49]. However, iNOS expression in broilers with PAH is controversial [49,50]{#2}. In the present analysis, INOS was up-regulated in pulmonary arteries from AS broilers, which lays a foundation for further research. Transforming growth factor-β (TGF-β) was reported to induce the overexpression of endothelin-1 and inflammatory cytokines, both of which stimulate vasoconstriction, proliferation, and remodeling, suggesting that the TGF-β signaling pathway may play a key role in the pathogenesis of PAH, both in humans and broilers [51,52]. It was reported that hypothermia and salt are linked to the observed gene expression results also to pulmonary artery thickening [53]. And in our study, the TGF-β family member BMP7 was down regulated in the pulmonary arteries from AS broiler caused by the cold temperature and salt water. Bone morphogenetic protein (BMP), a TGF- β family member, regulates transcription by phosphorylating Smads (Smad1/5/8). It has been widely reported that BMP4 mediates the process of pulmonary artery remodeling and is involved in the regulation of cell proliferation, migration, and intracellular calcium homeostasis[54]. Few studies have identified the role for BMP7 in AS progression, so our findings may provide another potential candidate gene for preventing PAH in humans and broilers.

Consistently, in this study, the pathological observation found the pulmonary artery remodeling, presented by thickening of the artery wall, especially in the medial intima consisting of vascular smooth muscle cells, and the more compact tunica adventitia (Fig 5). Previous pathological studies also reported similar characteristics of pulmonary artery remodeling in AS broilers, which resulted from hypertrophy, proliferation, and sparse migration of the affected cells (endothelial cells, smooth muscle cells, fibroblasts, inflammatory cells, and platelets) [55,56]. Pulmonary vascular remodeling was confirmed as an important pathological contributor to AS that leads to an increase in pulmonary vascular resistance, prolonging elevated arterial hypertension [24]. Among vascular well cells, the proliferation and hypertrophy of vascular smooth muscle cells was thought to be a main contributor that facilitates pulmonary vascular remodeling and has an important role in AS progression [57,58], this was in accord with the current changes in this study. Comprehensively, the pathological changes further supported the present RNA-seq findings in the pulmonary arteries from broilers with or without AS, and we revealed some specific components of complex molecular circuitry regulating pulmonary artery remodeling underlying in AS progression.

## Conclusion

The current study firstly provided the database of pulmonary arteries from broilers with and without ascites syndrome. Considering that the pulmonary artery remodeling plays a key step to the elevated pulmonary hypertension further causing the occurrence of PH in both human and broilers, our study further take a step to better understand the molecular mechanism of pulmonary artery remodeling in AS progression. We revealed specific pathways of the complex molecular circuitry of pulmonary artery remodeling in AS broilers like ribosome, Jak-STAT and NOD-like receptor signaling pathways which regulate pulmonary artery remodeling through vascular smooth cell proliferation, inflammation and vascular smooth cell proliferation together. Some notable DEGs within these pathways included downregulation of genes like RPL 5, 7, 8, 9, 14; upregulation of genes such as IL-6, K60, STAT3, STAT5 Pim1 and SOCS3; IKKα, IkB, P38, five cytokines IL-6, IL8, IL-1β, IL-18, and MIP-1β. Consistently, the pathological observation found the pulmonary artery remodeling in this study, which further demonstrate that revealed target genes and pathways in this study is meaningful for the prevention of PAH in both humans and broilers in the future.

## Supporting Information

S1 Fig Correlation coefficients of gene expression between the two biological replicate samples in the disease (D) and normal (N) groups. The R2 between D1 and D2 is close to 1, as is that between N1 and N2.
(DOCX)

S2 Fig Gene expression profiling. The gene expression density distribution between disease (D) and normal (N) samples shown in reads per kilobase per million reads (RPKM).
(DOCX)

S3 Fig Analysis of three main categories by GO enrichment. Red indicates up-regulated genes, while blue indicates down-regulated genes for a given GO term. The x-axis and y-axis indicate the subcategory name and gene number, respectively.
(DOCX)

S4 Fig Jak-STAT signaling pathway. Genes in red rectangle were upregulated were upregulated.
(DOCX)

S5 Fig NOD-like receptor signaling pathway. Genes in red rectangle were upregulated were upregulated.
(DOCX)

S1 Table Primers of selected genes for qRT-PCR. (DOCX)

S2 Table RPKM results of gene expression. (DOCX)

S3 Table The 13 DEGs that repeatedly enriched to 6 significantly enriched GO terms (DOCX)

S4 Table The top 20 enriched key pathways in the pulmonary arteries from AS and normal broilers. (DOCX)

S5 Table Putative genes related to ribosome with altered expression. (DOCX)

S6 Table Putative significantly differentially expressed molecules and genes enriched to the cytokine-cytokine receptor pathway. (DOCX)

S7 Table Putative significantly differential expressed molecules and genes enriched in immune response related pathways (DOCX)

S8 Table Putative significantly differential expressed molecules and genes enriched in fatty acid and ammo acid metabolism related pathways (DOCX)

S9 Table Putative significantly differential expressed molecules and genes enriched in pulmonary artery contraction related pathways. (DOCX)
