# Supplementary figures and images for: Transcriptome Analysis and Gene Identification in the Pulmonary Artery of Broilers with Ascites Syndrome
Source: PLoS One. 2016 Jun 8;11(6):e0156045. doi: 10.1371/journal.pone.0156045 (PMC4898705; doi:10.1371/journal.pone.0156045)

S1 Fig


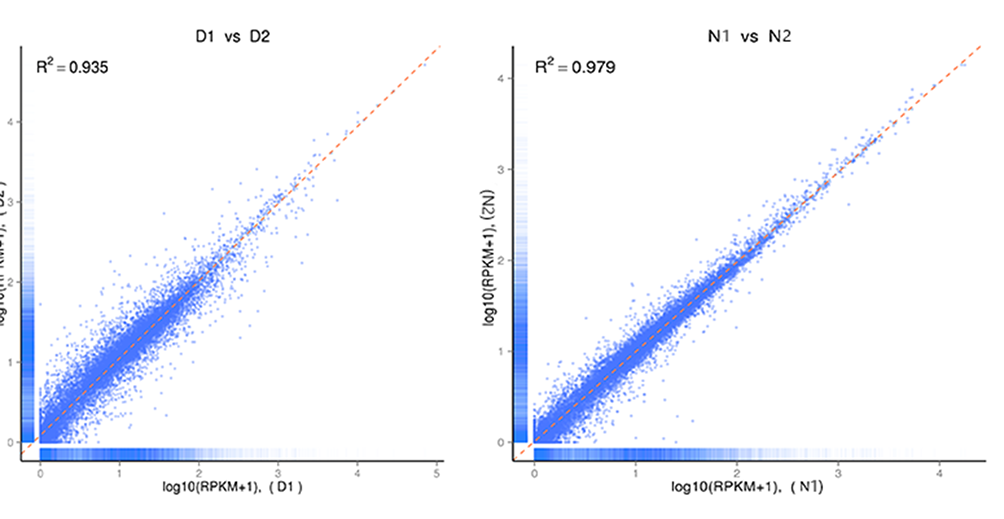

Supplement: S1 Fig — The R2 between D1 and D2 is close to 1, as is that between N1 and N2. (DOCX) [file pone.0156045.s001.docx]

S2 Fig


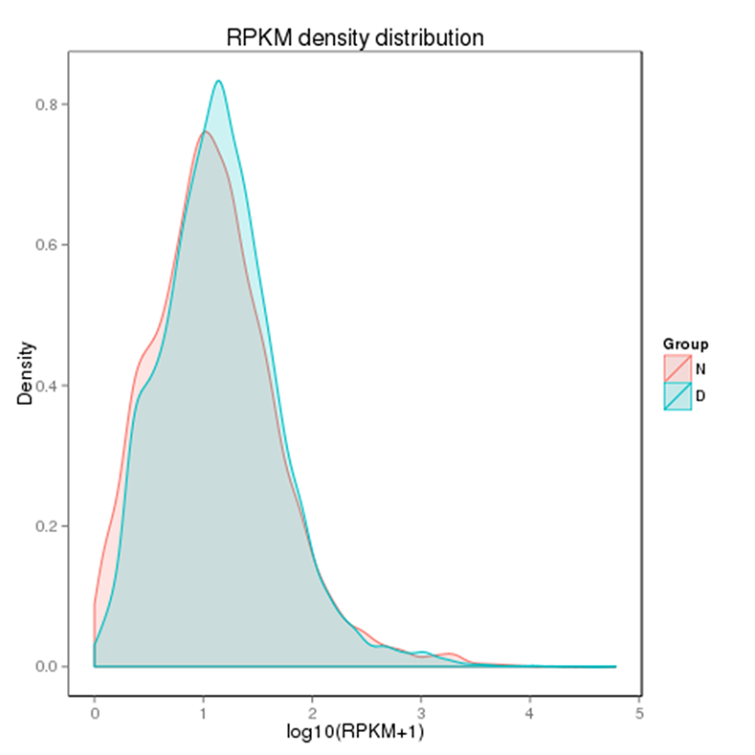

Supplement: S2 Fig — The gene expression density distribution between disease (D) and normal (N) samples shown in reads per kilobase per million reads (RPKM). (DOCX) [file pone.0156045.s002.docx]

S3 Fig


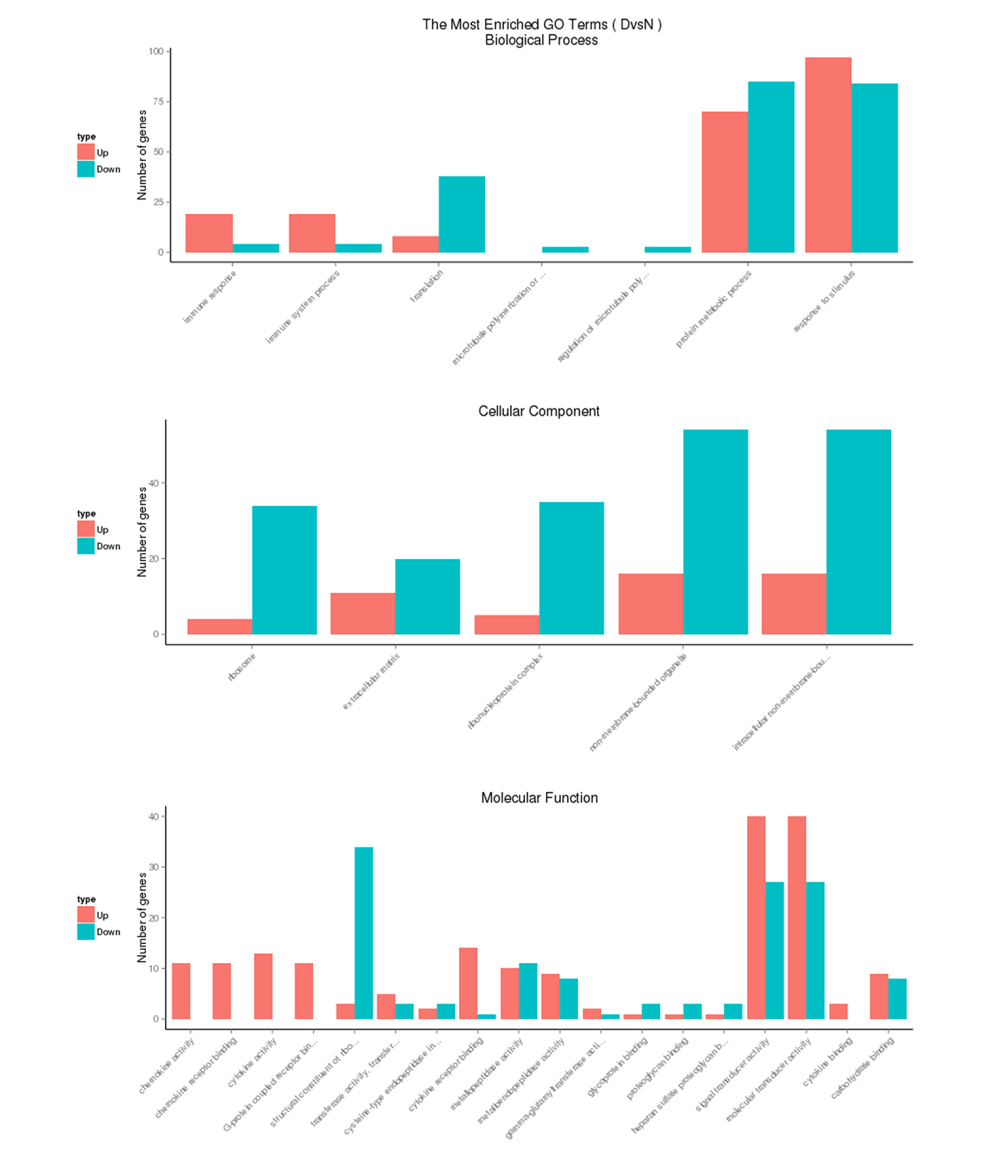

Supplement: S3 Fig — Red indicates up-regulated genes, while blue indicates down-regulated genes for a given GO term. The x-axis and y-axis indicate the subcategory name and gene number, respectively. (DOCX) [file pone.0156045.s003.docx]

S4 Fig


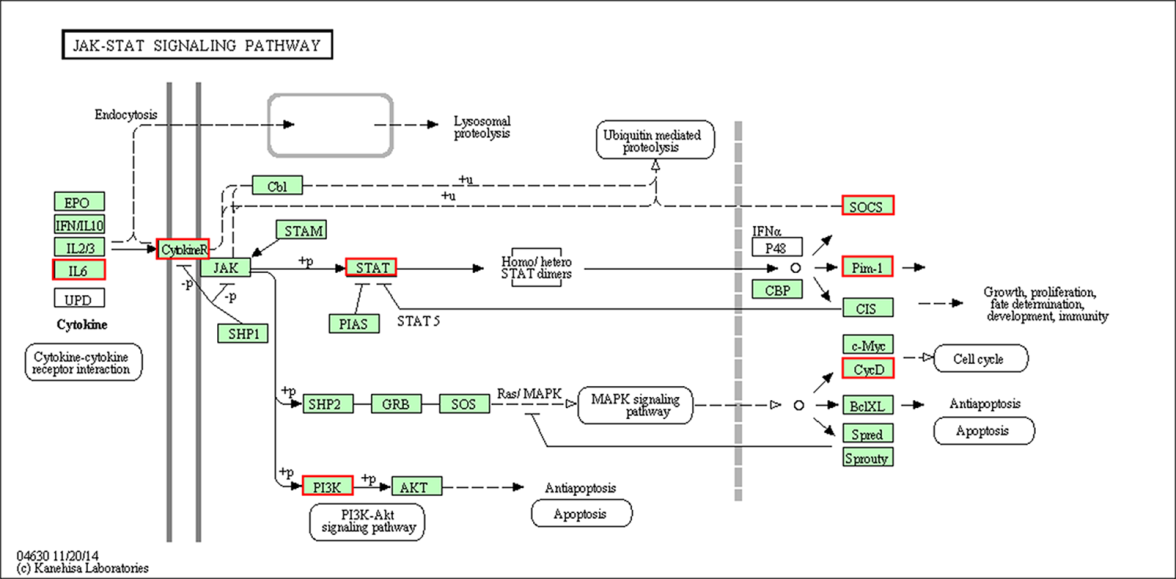

Supplement: S4 Fig — Genes in red rectangle were upregulated were upregulated. (DOCX) [file pone.0156045.s004.docx]

S5 Fig


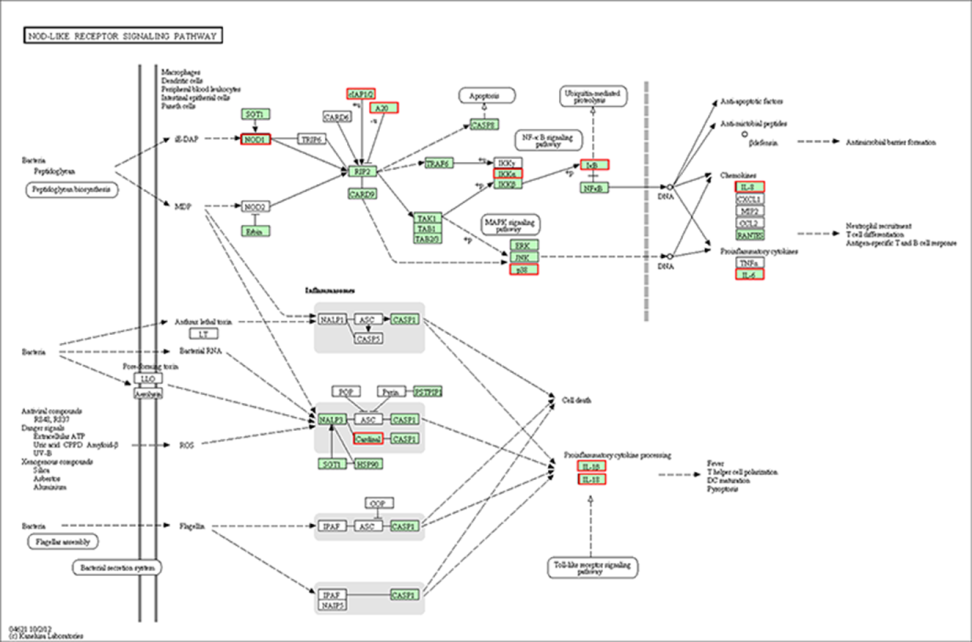

Supplement: S5 Fig — Genes in red rectangle were upregulated were upregulated. (DOCX) [file pone.0156045.s005.docx]
