# Supplementary material for: Transcriptome Analysis and Gene Identification in the Pulmonary Artery of Broilers with Ascites Syndrome
Source: PLoS One. 2016 Jun 8;11(6):e0156045. doi: 10.1371/journal.pone.0156045 (PMC4898705; doi:10.1371/journal.pone.0156045)
Supplement: S1 Table — (DOCX) [file pone.0156045.s006.docx]

**S1 Table Primers of selected genes for qRT-PCR.**

| **Gene** | **Forward primer** | **Reverse primer** |
| --- | --- | --- |
| CCL20 | 5' TCCCAGGCACAAAGCAACCAA 3' | 5' CCTCTTGAGCTTCTGGCTCAGG 3' |
| IL6 | 5' CGACGAGGAGAAATGCCTGAC 3' | 5' ACTTCAGATTGGCGAGGAGGG 3' |
| MAPK11 | 5' AAAGGGAAGGCCCTGTTTCCG 3' | 5' GATTGGCACCACGAAACACTGC 3' |
| IL2RG | 5' ACCATCCACAACATGAGCGGC 3' | 5' CAGCTGGGTGTTGCCACAATAG 3' |
| MX1 | 5' ACAGGAGAAAGGACGCTTGGG 3' | 5' TCTTGCTGGATTGCGGAGGTG 3' |
| GP130 | 5' TTCAGCAGGGTCGTCCTTCAA 3' | 5' GGATGGATCCACAGACACAGCTG 3' |
| TNFSF10 | 5' GGACGTGGTGGAAAGCAAAGAC 3' | 5' CCTGTCACCGTTGATTGCAGAC 3' |
| IL13RA1 | 5' CCTTCCCTCAGGCTGCCAATA 3' | 5' CCGATTCACTCCACTGCAGATG 3' |
| RPL8 | 5' CGGAATTGTTGCTGGTGGAGG 3' | 5' TGAAGGCTTGCCAATGTGCTG 3' |
| RPL27A | 5' CATCCTGGAGGCCGTGGTAAT 3' | 5' GCGCACAACATCGATGACTGG 3' |
| RPS24 | 5' CTTCAGCGCAAGCAGATGGTG 3' | 5' GCCACCACCAAAGTGAGTTCTG 3' |
| ACTB | 5' TGCTGTCCCTGTATGCCTCTGG 3' | 5' TGCTCGAAATCCAGTGCGACG 3' |
