# Supplementary material for: Transcriptome Analysis and Gene Identification in the Pulmonary Artery of Broilers with Ascites Syndrome
Source: PLoS One. 2016 Jun 8;11(6):e0156045. doi: 10.1371/journal.pone.0156045 (PMC4898705; doi:10.1371/journal.pone.0156045)
Supplement: S2 Table — (DOCX) [file pone.0156045.s007.docx]

**S2 Table RPKM results of gene expression**

| RPKM Interval | N2 | N3 | D1 | D2 |
| --- | --- | --- | --- | --- |
| 0~1 | 4936(31.83%) | 4992(32.19%) | 4876(31.44%) | 4404(28.40%) |
| 1~3 | 1560(10.06%) | 1459(9.41%) | 1460(9.41%) | 1320(8.51%) |
| 3~15 | 4549(29.33%) | 4575(29.50%) | 4568(29.46%) | 4536(29.25%) |
| 15~60 | 3181(20.51%) | 3259(21.01%) | 3401(21.93%) | 3991(25.74%) |
| >60 | 1282(8.27%) | 1223(7.89%) | 1203(7.76%) | 1257(8.11%) |
