# Supplementary material for: Transcriptome Analysis and Gene Identification in the Pulmonary Artery of Broilers with Ascites Syndrome
Source: PLoS One. 2016 Jun 8;11(6):e0156045. doi: 10.1371/journal.pone.0156045 (PMC4898705; doi:10.1371/journal.pone.0156045)
Supplement: S3 Table — (DOCX) [file pone.0156045.s008.docx]

**S3 Table The 13 DEGs that repeatedly enriched to 6 significantly enriched GO terms.**

| **Gene name** | **Gene**  **ID** | **Readcount D** | **Readcount N** | **Description** | **Padj** |
| --- | --- | --- | --- | --- | --- |
| IL6 | ENSGALG00000010915 | 937.8956876 | 8.124493906 | Interleukin-6 | 2.23E-06 |
| IL8 | ENSGALG00000026098 | 1281.912859 | 3.835431728 | Chemokineinterleukin-8-like domain | 5.93E-47 |
| K60 | ENSGALG00000011668 | 469.7347847 | 0 | Chemokine interleukin-8-like domain | 3.31E-21 |
| CSF3 | ENSGALG00000026420 | 61.87813998 | 1.113649027 | Interleukin-6/Interleukin-23/GCSF/MGF | 0.00010085 |
| CCL4 | ENSGALG00000000956 | 677.900518 | 12.08460536 | Chemokine interleukin-8-like domain | 3.48E-15 |
| CXCL13L2 | ENSGALG00000010338 | 103.1519077 | 0.9072609 | Chemokine interleukin-8-like domain\|\|CXC chemokine | 1.15E-10 |
| CCL20 | ENSGALG00000003003 | 132.2897838 | 1.010454963 | Chemokine interleukin-8-like domain | 2.70E-13 |
| - | ENSGALG00000027051 | 351.0768539 | 24.62284118 | Chemokine interleukin-8-like domain | 0.01767 |
| - | ENSGALG00000010336 | 71.10067384 | 0.45363045 | Chemokine interleukin-8-like domain\|\|CXC chemokine | 4.47E-08 |
| JSC | ENSGALG00000006346 | 253.5901249 | 0.9072609 | Chemokine interleukin-8-like domain | 0.016737 |
| CX3CL1 | ENSGALG00000026663 | 599.4907094 | 162.4747439 | Chemokine interleukin-8-like domain | 0.043937 |
| IL-1BETA | ENSGALG00000000534 | 423.770755 | 15.92003709 | Interleukin-1 alpha/beta | 1.86E-18 |
| TNFSF10 | ENSGALG00000009179 | 1013.635787 | 327.5701935 | Tumour necrosis factor domain | 0.008668 |

**Note:** A gene with a Padj<0.05 is considered as significantly differential expressed. Padj means the corrected-P value；Readcount D means the readcounts of disease samples; Readcount N means the readcounts of normal samples.
