# Supplementary material for: Transcriptome Analysis and Gene Identification in the Pulmonary Artery of Broilers with Ascites Syndrome
Source: PLoS One. 2016 Jun 8;11(6):e0156045. doi: 10.1371/journal.pone.0156045 (PMC4898705; doi:10.1371/journal.pone.0156045)
Supplement: S4 Table — (DOCX) [file pone.0156045.s009.docx]

**S4 Table The top 20 enriched key pathways in the pulmonary arteries from AS and normal broilers.**

| **KEGG Pathway** | **N0. of Genes** | **P-value** | **Padj** |
| --- | --- | --- | --- |
| Ribosome | 40 | 1.9296165983e-12 | 2.35413224992e-10 |
| NOD-like receptor signaling pathway | 13 | 2.60866902426e-05 | 0.00106085873653 |
| Cytokine-cytokine receptor interaction pathway | 35 | 1.23307974926e-07 | 7.5217864705e-06 |
| Jak-STAT signaling pathway | 22 | 0.000879999990873 | 0.0268399997216 |
| Influenza A | 22 | 0.00137927933874 | 0.0336544158653 |
| Adipocytokine signaling pathway | 14 | 0.00334072705908 | 0.067928116868 |
| Metabolism of xenobiotics by cytochrome P450 | 8 | 0.00708610239632 0.123500641764 | 0.123500641764 |
| Toll-like receptor signaling pathway | 14 | 0.00989898930217 | 0.150959586858 |
| RIG-I-like receptor signaling pathway | 10 | 0.0178701990438 | 0.203481249443 |
| vascular smooth muscle contraction pathways | 14 | 0.0187804363851 | 0.203481249443 |
| Alanine, aspartate and glutamate metabolism | 7 | 0.0205040714527 | 0.203481249443 |
| Cytosolic DNA-sensing pathway | 8 | 0.0208989004061 | 0.203481249443 |
| Salmonella infection | 11 | 0.0223879264896 | 0.203481249443 |
| [Cysteine and methionine metabolism](F:/%E4%B8%B4%E5%BA%8A%20Fighting/8.DEG_KEGGEnrichment/8.3.DEG_KEGGPath/ALL/DvsN/src/gga00270.html) | 7 | 0.0233503073131 | 0.203481249443 |
| Herpes simplex infection | 19 | 0.0252808140371 | 0.205617287501 |
| Apoptosis | 10 | 0.0585036696714 | 0.446090481244 |
| Pyruvate metabolism | 6 | 0.066379152698 | 0.47636803701 |
| Steroid hormone biosynthesis | 6 | 0.0732228888935 | 0.496288469167 |
| Glutathione metabolism | 6 | 0.0804542767036 | 0.51660114515 |
| Fatty acid biosynthesis | 3 | 0.104933575317 | 0.609111181186 |

**Note:** “NO.of genes” means the the number of DEGs enriched in corresponding pathways. “Padj” represents the corrected P-value. Padj<0.05 means this function was significantly enriched.
