# Supplementary material for: Transcriptome Analysis and Gene Identification in the Pulmonary Artery of Broilers with Ascites Syndrome
Source: PLoS One. 2016 Jun 8;11(6):e0156045. doi: 10.1371/journal.pone.0156045 (PMC4898705; doi:10.1371/journal.pone.0156045)
Supplement: S5 Table — (DOCX) [file pone.0156045.s010.docx]

**Table S5 Putative genes related to ribosome with altered expression**

| **Gene Name** | **Gene ID** | **Readcount**  **D** | **Readcount**  **N** | **Padj** | **Description** |
| --- | --- | --- | --- | --- | --- |
| RPL3L | ENSGALG00000012172 | 8515.066838 | 20636.35141 | 0.0050846 | Translation protein, beta-barrel domain |
| RPL5 | ENSGALG00000005922 | 6389.700326 | 14604.23788 | 0.010247 | Ribosomal protein L18/L5 |
| RPL7 | ENSGALG00000015637 | 6389.700326 | 14604.23788 | 0.0047981 | Ribosomal protein L30 |
| RPL8 | ENSGALG00000016232 | 6909.142215 | 19424.04571 | 0.00058216 | - |
| RPL9 | ENSGALG00000000150 | 5095.234138 | 11206.42372 | 0.017803 | Ribosomal protein L6 |
| RPL10A | ENSGALG00000002644 | 4029.214337 | 9143.100091 | 0.012356 | Ribosomal protein L1biogenesis protein |
| RPL14 | ENSGALG00000011523 | 4421.868794 | 10576.74231 | 0.0057521 | Ribosomal protein L14 |
| RPL21 | ENSGALG00000027035 | 5561.831907 | 13575.09992 | 0.0040968 | Ribosomal protein L21e |
| RPL22 | ENSGALG00000000719 | 2436.996693 | 5641.892697 | 0.0089209 | Ribosomal protein L22e |
| RPL23A | ENSGALG00000003966 | 4277.186466 | 9280.040499 | 0.021685 | Ribosomal protein L23 |
| - | ENSGALG00000022174 | 0.54178759 | 509.759552 | 7.84E-36 | Ribosomal protein L22/L17 |
| RPL23 | ENSGALG00000001634 | 4633.486126 | 9624.669784 | 0.033901 | Ribosomal protein L14b/L23e |
| RPL24 | ENSGALG00000015339 | 3291.922356 | 6597.854215 | 0.048241 | Ribosomal protein L24e-related |
| RPL26 | ENSGALG00000002868 | 5122.620904 | 11764.92569 | 0.0096719 | Ribosomal protein L26/L24P |
| RPL27A | ENSGALG00000005948 | 3774.053878 | 9569.858602 | 0.0026487 | - |
| RPL29 | ENSGALG00000026978 | 3882.868814 | 8973.402392 | 0.0090557 | Ribosomal protein L29e |
| RPL30 | ENSGALG00000008212 | 2220.612576 | 5161.638493 | 0.009915 | 50S ribosomal protein L30e-like |
| - | ENSGALG00000028992 | 1715.715229 | 4150.119203 | 0.0062824 | Ribosomal protein L34Ae |
| RPL35 | ENSGALG00000001039 | 2506.244692 | 5305.041704 | 0.026631 | Ribosomal protein L29 |
| RPL37A | ENSGALG00000011472 | 6109.212345 | 14907.49544 | 0.0041341 | Ribosomal protein L37ae |
| RPL38 | ENSGALG00000001465 | 1898.772262 | 4070.207633 | 0.022197 | Ribosomal protein L38e |
| RPL39 | ENSGALG00000008620 | 3285.351583 | 6845.476533 | 0.028664 | Ribosomal protein L39e domain |
| RPS3A | ENSGALG00000010077 | 12280.69748 | 25309.65535 | 0.034287 | Ribosomal protein S3Ae |
| RPS3 | ENSGALG00000017330 | 6222.130078 | 12716.95007 | 0.038601 | Ribosomal protein S3, C-terminal |
| RPS4 | ENSGALG00000004831 | 7193.717947 | 15036.97498 | 0.030897 | Ribosomal protein S4e |
| MRPS6 | ENSGALG00000027579 | 214.8394687 | 517.5593294 | 0.026844 | Ribosomal protein S6 |
| RPS7 | ENSGALG00000016392 | 5003.84432 | 11726.14134 | 0.0079056 | Ribosomal protein S7e |
| RPS8 | ENSGALG00000010124 | 9943.31329 | 21950.09929 | 0.014629 | Ribosomal protein S8e |
| RPS10 | ENSGALG00000002813 | 5118.957793 | 11327.84502 | 0.015946 | Plectin/S10, N-terminal |
| RPS12 | ENSGALG00000013990 | 5221.69366 | 13866.44806 | 0.0013843 | Ribosomal protein S12e |
| RPS15A | ENSGALG00000006771 | 4466.575567 | 9847.336075 | 0.015586 | Ribosomal protein S8 |
| RPS16 | ENSGALG00000026490 | 4466.575567 | 9847.336075 | 0.0028675 | Ribosomal protein S9 |
| RPS17 | ENSGALG00000002157 | 2449.817316 | 5024.775874 | 0.037337 | Ribosomal protein S17e |
| RPS21 | ENSGALG00000005338 | 1935.50086 | 4065.819925 | 0.027829 | Ribosomal protein S21e |
| RPS24 | ENSGALG00000004871 | 1935.50086 | 4065.819925 | 0.00018071 | Ribosomal protein S24e |
| RPS25 | ENSGALG00000007699 | 5127.357596 | 11581.10428 | 0.012523 | Ribosomal protein S25 |
| RPS28 | ENSGALG00000024398 | 3049.143694 | 6376.854163 | 0.030841 | Ribosomal protein S28e |

**Note:** A gene with a Padj<0.05 is considered as significantly differential expressed. Padj means the corrected-P value；Readcount D means the readcounts of disease samples; Readcount N means the readcounts of normal samples.
