# Supplementary material for: Transcriptome Analysis and Gene Identification in the Pulmonary Artery of Broilers with Ascites Syndrome
Source: PLoS One. 2016 Jun 8;11(6):e0156045. doi: 10.1371/journal.pone.0156045 (PMC4898705; doi:10.1371/journal.pone.0156045)
Supplement: S6 Table — (DOCX) [file pone.0156045.s011.docx]

**S6 Table Putative significantly differential expressed molecules and genes enriched in** **Cytokine-cytokine receptor pathway.**

| **Signaling molecules** | **Gene name** | **Gene ID** | **Padj** | **Description** |
| --- | --- | --- | --- | --- |
| IL8 | K60 | ENSGALG00000011668 | 3.31E-21 | Chemokine interleukin-8-like domain |
|  | IL8 | ENSGALG00000026098 | 5.93E-47 | Chemokine interleukin-8-like domain |
| CXCL13 | CXCL13L2 | ENSGALG00000010338 | 1.15E-10 | Chemokine interleukin-8-like domain |
|  | - | ENSGALG00000010336 | 4.47E-08 | Chemokine interleukin-8-like domain |
| CXCL14 | JSC | ENSGALG00000006346 | 0.016737 | Chemokine interleukin-8-like domain |
| CX3CL1 | CX3CL1 | ENSGALG00000026663 | 0.043937 | Chemokine interleukin-8-like domain |
| CCL20 | CCL20 | ENSGALG00000003003 | 2.70E-13 | Chemokine interleukin-8-like domain |
| CCL4 | CCL4 | ENSGALG00000000951 | 3.09E-25 | Chemokine interleukin-8-like domain |
| CCR5 | CCR2 | ENSGALG00000011733 | 0.023594 | CC chemokine receptor 2\|\|7TM GPCR |
| IL6 | IL6 | ENSGALG00000010915 | 2.23E-06 | Interleukin-6/Interleukin-23 |
| IL6ST | GP130 | ENSGALG00000014716 | 0.013903 | Immunoglobulin C2-set-like, ligand-binding |
| IL4R | CCR2 | ENSGALG00000011733 | 0.023594 | CC chemokine receptor 2 |
| IL13RA1 | IL13RA1 | ENSGALG00000006032 | 0.022197 | Interleukin-6 receptor alpha, binding |
| IL12RB1 | IL12RB1 | ENSGALG00000027301 | 0.028163 | Fibronectin, type III |
| IL2RG | IL2RG | ENSGALG00000005638 | 0.0013193 | Interleukin-6 receptor alpha, binding |
|  | IL4RA | ENSGALG00000006313 | 0.02562 | Interleukin-4 receptor alpha |
| CSF3 | CSF3 | ENSGALG00000026420 | 0.00010085 | Interleukin-6/Interleukin-23/GCSF/MGF |
|  | CSF3R | ENSGALG00000002112 | 1.54E-10 | Immunoglobulin C2-set-like, ligand-binding |
| HGF | HGF/SF | ENSGALG00000008461 | 0.00039565 | Hepatocyte growth factor |
| GSF1 | CSF1 | ENSGALG00000028217 | 0.033637 | Four-helical cytokine-like, core |
| IFNGR1 | CSF1 | ENSGALG00000028217 | 0.033637 | Four-helical cytokine-like, core |
| IL10RA | IL10R1 | ENSGALG00000024075 | 0.00027589 | Interferon alpha/beta receptor |
| IL10RB | IL10R2 | ENSGALG00000015941 | 0.024509 | Interferon alpha/beta receptor, beta chain |
| IL20RA | IL20RA | ENSGALG00000013869 | 4.37E-13 | Interferon alpha/beta receptor, beta chain |
| IL22RA | IL22RA2 | ENSGALG00000013868 | 0.038518 | Interferon alpha/beta receptor, beta chain |
| TNFSF10 | TNFSF10 | ENSGALG00000009179 | 0.008668 | Tumour necrosis factor ligand |
| SF11A | TNFRSF11A | ENSGALG00000012891 | 0.047899 | Tumour necrosis factor receptor 11A |
| SF21 | TNFRSF21 | ENSGALG00000016719 | 2.59E-05 | Tumour necrosis factor receptor 21 |
| SF1B | TNFRSF1B | ENSGALG00000004419 | 0.047849 | TNFR/NGFR cysteine-rich region |
| SF1A | TNFRSF1A | ENSGALG00000014890 | 0.00093911 | TNFR/NGFR cysteine-rich region |
| BM7 | BMP7 | ENSGALG00000007668 | 0.00706 | Transforming growth factor-beta |
| IL1B | IL-1BETA | ENSGALG00000000534 | 1.86E-18 | Cytokine, Interleukin-1 alpha/beta |
|  | IL2RG | ENSGALG00000005638 | 0.0013193 | Interleukin-6 receptor alpha |
| IL1RAP | IL1RAP | ENSGALG00000007253 | 1.74E-05 | Interleukin-1 receptor type I/II |
| IL1R2 | IL1R2 | ENSGALG00000016782 | 5.64E-28 | Interleukin-1 receptor type domain |
| IL18 | IL18 | ENSGALG00000007874 | 0.020274 | Interleukin-18\|\|Cytokine, IL-1-like |

**Note:** A gene with a Padj<0.05 is considered as significantly differential expressed. Padj means the corrected-P value.
