# Supplementary material for: Transcriptome Analysis and Gene Identification in the Pulmonary Artery of Broilers with Ascites Syndrome
Source: PLoS One. 2016 Jun 8;11(6):e0156045. doi: 10.1371/journal.pone.0156045 (PMC4898705; doi:10.1371/journal.pone.0156045)
Supplement: S7 Table — (DOCX) [file pone.0156045.s012.docx]

**S7 Table** **Putative significantly differential expressed molecules and genes enriched in immune response related pathways**

| **Signaling molecus** | **Gene name** | **Gene ID** | **Padj** | **Description** | |
| --- | --- | --- | --- | --- | --- |
| **Toll-like receptor signaling pathway** | | | | | |
| PI3K | PIK3R5 | ENSGALG00000021573 | 2.22E-05 | | Phosphoinositide 3-kinase regulatory subunit 5/6 |
| IKKα | CHUK | ENSGALG00000003289 | 0.0019618 | | Serine/threonine/dual specificity protein kinase |
| TPL2 | MAP3K8 | ENSGALG00000007356 | 1.04E-12 | | Serine-threonine/tyrosine-protein kinase |
| OPN | SPP1 | ENSGALG00000010926 | 0.031019 | | Osteopontin |
| IF7 | IRF-3 | ENSGALG00000014297 | 4.68E-05 | | Interferon regulatory factor-3 |
| P38 | MAPK11 | ENSGALG00000008612 | 0.0003684 | | Mitogen-activated protein (MAP) kinase, p38 |
| IﻻBβ | NFKBIA | ENSGALG00000027864 | 0.046652 | | Ankyrin repeat-containing domain |
| IL-1β | IL-1BETA | ENSGALG00000000534 | 1.86E-18 | | Cytokine, Interleukin-1 alpha/beta |
| IL6 | IL6 | ENSGALG00000010915 | 2.23E-06 | | Interleukin-6/Interleukin-23 |
| IL8 | IL8 | ENSGALG00000026098 | 5.93E-47 | | Chemokine interleukin-8-like domain |
|  | K60 | ENSGALG00000011668 | 3.31E-21 | | Chemokine interleukin-8-like domain |
| MIP-1β | CCL4 | ENSGALG00000000951 | 3.09E-25 | | Chemokine interleukin-8-like domain |
| **RIG-I-like receptor signaling pathway** | | | | | |
| TRIM25 | TRIM25 | ENSGALG00000003144 | 0.008668 | | Zinc finger, C3HC4 |
| LGP2 | DHX58 | ENSGALG00000023821 | 1.56E-06 | | P-loop containing nucleoside triphosphate hydrolase |
| TRAF2 | TRAF2 | ENSGALG00000009014 | 0.018489 | | TNF receptor-associated factor TRAF |
| IKK | IKBKE | ENSGALG00000013356 | 1.98E-15 | | Serine/threonine/dual specificity protein kinase |
|  | IRF-3 | ENSGALG00000014297 | 4.68E-05 | | Interferon regulatory factor-3 |
| IKKα | CHUK | ENSGALG00000003289 | 0.0019618 | | Serine/threonine/dual specificity protein kinase |
| P38 | MAPK11 | ENSGALG00000008612 | 0.0003684 | | Mitogen-activated protein (MAP) kinase, p38 |
| I B | NFKBIA | ENSGALG00000027864 | 0.046652 | | Ankyrin repeat-containing domain |
| IL8 | IL8 | ENSGALG00000026098 | 5.93E-47 | | Chemokine interleukin-8-like domain |
|  | K60 | ENSGALG00000011668 | 3.31E-21 | | Chemokine interleukin-8-like domain |
| **MAPK signaling pathway** | | | | | |
| NT3/4 | NTF3 | ENSGALG00000027299 | 0.0019437 | | Nerve growth factor-related |
| TrkA/B | NTRK2 | ENSGALG00000012594 | 0.044303 | | Immunoglobulin subtype |
| Pl20GAF | - | ENSGALG00000022611 | 5.64E-08 | | Rho GTPase activation protein |
| PTP | DUSP3 | ENSGALG00000029003 | 0.042215 | | Protein-tyrosine phosphatase, receptor |
| PTP | PTPN5 | ENSGALG00000006368 | 0.0070559 | | Protein-tyrosine phosphatase, receptor |
| IKK | CHUK | ENSGALG00000003289 | 0.0019618 | | Serine/threonine/dual specificity protein kinase |
| MKP | DUSP5 | ENSGALG00000008581 | 0.00019289 | | Protein-tyrosine phosphatase-like |
|  | DUSP4 | ENSGALG00000011419 | 0.031055 | | Protein-tyrosine phosphatase-like |
| NFﻻK | NFKB2 | ENSGALG00000005653 | 5.54E-07 | | nuclear factor of kappa light polypeptide gene enhancer in B-cells 2 |
| IL-1 | IL-1BETA | ENSGALG00000000534 | 1.86E-18 | | Cytokine, Interleukin-1 alpha/beta |
| TNFR | TNFRSF1A | ENSGALG00000014890 | 0.00093911 | | TNFR/NGFR cysteine-rich region |
| IL1R | IL1R1 | ENSGALG00000016783 | 0.00081311 | | Toll/interleukin-1 receptor |
|  | IL1R2 | ENSGALG00000016782 | 5.64E-28 | | Interleukin-1 receptor type |
| TRAF2 | TRAF2 | ENSGALG00000009014 | 0.018489 | | TNF receptor-associated factor TRAF |
| GADD45 | GADD45 | ENSGALG00000028005 | 0.010498 | | 50S ribosomal protein L30e-like |
| Tpl2/cot | MAP3K8 | ENSGALG00000007356 | 1.04E-12 | | Serine-threonine/tyrosine-protein kinase |
| ASK2 | MAP3K6 | ENSGALG00000028785 | 3.54E-11 | | Serine/threonine/dual specificity protein kinase |
| P38 | MAPK11 | ENSGALG00000008612 | 0.0003684 | | Serine-threonine/tyrosine-protein kinase |

**Note:** A gene with a Padj<0.05 is considered as significantly differential expressed. Padj means the corrected-P value.
