# Supplementary material for: Transcriptome Analysis and Gene Identification in the Pulmonary Artery of Broilers with Ascites Syndrome
Source: PLoS One. 2016 Jun 8;11(6):e0156045. doi: 10.1371/journal.pone.0156045 (PMC4898705; doi:10.1371/journal.pone.0156045)
Supplement: S8 Table — (DOCX) [file pone.0156045.s013.docx]

**S8 Table Putative significantly differential expressed molecules and genes enriched in fatty acid and ammo acid metabolism related pathways.**

| **Signaling molecules** | **Gene name** | | **Gene ID** | **Padj** | **State** | **Description** |
| --- | --- | --- | --- | --- | --- | --- |
| **Fatty acid biosynthesis** | | | | | | |
|  | ACSL1 | | ENSGALG00000010628 | 9.63E-20 | up | AMP-dependent synthetase/ligase |
|  | ACSBG2 | | ENSGALG00000001749 | 3.48E-05 | up | AMP-dependent synthetase/ligase |
|  | ACSL5 | | ENSGALG00000008840 | 0.021926 | up | AMP-dependent synthetase/ligase |
| **Fatty acid degradation** | | | | | | |
|  | ALDH7A1 | | ENSGALG00000008229 | 0.01793 | up | Aldehyde/histidinol dehydrogenase |
|  | ACSL1 | | ENSGALG00000010628 | 9.63E-20 | up | AMP-dependent synthetase/ligase |
|  | ACSBG2 | | ENSGALG00000001749 | 3.48E-05 | up | AMP-dependent synthetase/ligase |
|  | ACSL5 | | ENSGALG00000008840 | 0.021926 | up | AMP-dependent synthetase/ligase |
| **Fatty acid metabolism** | | | | | | |
|  | ACSBG2 | | ENSGALG00000001749 | 3.48E-05 | up | AMP-dependent synthetase/ligase |
|  | SCD | | ENSGALG00000005739 | 8.09E-08 | up | Fatty acid desaturase, type 1, core |
|  | ACSL5 | | ENSGALG00000008840 | 0.021926 | up | AMP-dependent synthetase/ligase |
|  | ACSL1 | | ENSGALG00000010628 | 9.63E-20 | up | AMP-dependent synthetase/ligase |
| **Adipocytokine signaling pathway** | | | | | | |
| TNFR1 | TNFRSF1A | ENSGALG00000014890 | | 0.000939 | up | TNFR/NGFR cysteine-rich region |
| TNFR2 | TNFRSF1B | ENSGALG00000004419 | | 0.047849 | up | TNFR/NGFR cysteine-rich region |
| TNAF2 | TRAF2 | ENSGALG00000009014 | | 0.018489 | up | \|TNF receptor-associated factor TRAF |
| IKK | CHUK | ENSGALG00000003289 | | 0.001961 | up | Serine/threonine/dual specificity protein kinase |
| IﻻK | NFKBIA | ENSGALG00000027864 | | 0.046652 | up | Ankyrin repeat-containing domain |
|  | NFKBIE | ENSGALG00000010171 | | 0.001176 | up | Ankyrin repeat-containing domain |
| SOCS3 | SOCS3 | ENSGALG00000027786 | | 4.37E-12 | up | \|SOCS protein, C-terminal |
| STAT3 | STAT3 | ENSGALG00000003267 | | 0.000264 | up | STAT transcription facto |
| PGC1α | PPARGC1A | ENSGALG00000014398 | | 0.02534 | up | RNA recognition motif domain |
| FACS | ACSBG2 | ENSGALG00000001749 | | 3.48E-05 | up | AMP-dependent synthetase/ligase |
|  | ACSL5 | ENSGALG00000008840 | | 0.021926 | up | AMP-dependent synthetase/ligase |
|  | ACSL1 | ENSGALG00000010628 | | 9.63E-20 | up | AMP-dependent synthetase/ligase |
| NPY | NPY | ENSGALG00000010983 | | 3.04E-12 | down | Pancreatic hormone-like |
| **Peroxisome** | | | | | | |
| ACSL | ACSL1 | ENSGALG00000010628 | | 9.63E-20 | up | AMP-dependent synthetase/ligase |
|  | ACSL5 | ENSGALG00000008840 | | 0.021926 | up | AMP-dependent synthetase/ligase |
| INOS | INOS | ENSGALG00000005693 | | 3.54E-36 | up | Nitric oxide synthase, 1 |
| CAT | CAT | ENSGALG00000014471 | | 0.001857 | down | Catalase immune-responsive domain |
| DDO | DDO | ENSGALG00000015057 | | 7.31E-05 | down | FAD dependent oxidoreductase |
|  | GPX1 | ENSGALG00000028204 | | 0.025465 |  | Glutathione peroxidase |
| **Arachidonic acid metabolism** | | | | | | |
|  | TBXAS1 | ENSGALG00000012791 | | 0.023839 | up | Cytochrome P450, B-class, E-class |
|  | GPX1 | ENSGALG00000028204 | | 0.025465 | up | Glutathione peroxidase |
| CYP2G | - | ENSGALG00000011394 | | 6.29E-06 | up | Cytochrome P450, E-class, group I |
|  | PTGDS | ENSGALG00000028529 | | 1.27E-05 | up | Neutrophil gelatinase-associated lipocalin Prostaglandin D synthase |
| **Tryptophan metabolism** | | | | | | |
|  | ALDH7A1 | | ENSGALG00000008229 | 0.01793 | up | Aldehyde/histidinol dehydrogenase |
|  | IL4I1 | | ENSGALG00000000081 | 1.94E-51 | up | Flavin amine oxidase |
|  | - | | ENSGALG00000004501 | 3.22E-10 | up | Cytochrome P450, B-class |
|  | CYP1B1 | | ENSGALG00000025822 | 0.003082 | down | Cytochrome P450, E-classs |
|  | CAT | | ENSGALG00000014471 | 0.001857 | down | Catalase-like domain |
| **Arginine and Proline metabolism** | | | | | | |
|  | - | | ENSGALG00000008518 | 0.024001 | up | Glutamine synthetase, beta-Grasp |
|  | CRYD2 | | ENSGALG00000002576 | 0.010418 | up | L-Aspartase-like\|\|Fumarate lyase family |
|  | INOS | | ENSGALG00000005693 | 3.54E-36 | up | Nitric oxide synthase |
|  | - | | ENSGALG00000007728 | 0.034305 | up | FAD-linked oxidoreductase-like |
|  | ALDH7A1 | | ENSGALG00000008229 | 0.01793 | up | Aldehyde/histidinol dehydrogenase |
| [**Cysteine and methionine metabolism**](F:/%E4%B8%B4%E5%BA%8A%20Fighting/8.DEG_KEGGEnrichment/8.3.DEG_KEGGPath/ALL/DvsN/src/gga00270.html) | | | | | | |
|  | LDHA | | ENSGALG00000006300 | 0.000907 | up | Lactate dehydrogenase/glycoside hydrolase |
|  | LDHB | | ENSGALG00000013257 | 0.003957 | down | L-lactate/malate dehydrogenase\|\|L-lactate dehydrogenase |
|  | CDO1 | | ENSGALG00000002358 | 0.027097 | down | Cysteine dioxygenase type I |
|  | - | | ENSGALG00000004518 | 0.000785 | down | Betaine-homocysteine S-methyltransferase |
|  | MAT1A | | ENSGALG00000002479 | 0.029704 | down | S-adenosylmethionine synthetase |
|  | IL4I1 | | ENSGALG00000000081 | 1.94E-51 | up | Flavin amine oxidase |
|  | DNMT1 | | ENSGALG00000028997 | 0.038518 | up | DNA (cytosine-5)-methyltransferase 1 |
| **Alanine, aspartate and glutamate metabolism** | | | | | | |
|  | CRYD2 | | ENSGALG00000002576 | 0.010418 | up | L-Aspartase-like\|\|Fumarate lyase family |
|  | ASPA | | ENSGALG00000004669 | 0.027757 | up | Succinylglutamate desuccinylase/aspartoacylase |
|  | ASNS | | ENSGALG00000009748 | 0.043647 | up | Class II glutamine amidotransferase domain |
|  | FOLH1 | | ENSGALG00000017234 | 0.030559 | up | Protease-associated domain, PA |
|  | IL4I1 | | ENSGALG00000000081 | 1.94E-51 | up | Flavin amine oxidase |
|  | - | | ENSGALG00000008518 | 0.024001 | up | Glutamine synthetase, beta-Grasp |
|  | DDO | | ENSGALG00000015057 | 7.31E-05 | down | FAD dependent oxidoreductase |

**Note:** A gene with a Padj<0.05 is considered as significantly differential expressed. Padj means the corrected-P value.
