# Supplementary material for: Transcriptome Analysis and Gene Identification in the Pulmonary Artery of Broilers with Ascites Syndrome
Source: PLoS One. 2016 Jun 8;11(6):e0156045. doi: 10.1371/journal.pone.0156045 (PMC4898705; doi:10.1371/journal.pone.0156045)
Supplement: S9 Table — (DOCX) [file pone.0156045.s014.docx]

**Table S9 Putative significantly expressed genes enriched in pulmonary artery contraction related pathways.**

| **Signaling molecular** | **Gene name** | **Gene ID** | **Padj** | **state** | **Description** |
| --- | --- | --- | --- | --- | --- |
| **Vascular smooth muscle contraction** | | | | | |
| Kca | - | ENSGALG00000027617 | 0.0016541 | down | Potassium channel, calcium-activated |
|  | CO6 | ENSGALG00000002118 | 1.42E-05 | down | Potassium channel, calcium-activated |
| ADRA1 | ADRA1D | ENSGALG00000015974 | 3.10E-08 | down | G protein-coupled receptor |
|  | ADRA1B | ENSGALG00000001416 | 2.33E-05 | down | G protein-coupled receptor |
| MLCK | MYLK | ENSGALG00000011708 | 8.22E-05 | dowm | myosin-light-chain kinase |
| CaM | CALM | ENSGALG00000010023 | 0.028664 | down | Ca insensitivecalmodulin |
| PLC | PLCB4 | ENSGALG00000008907 | 5.15E-05 | down | Phosphatidylinositol-4, 5-bisphosphate phosphodiesterase beta |
| CaD | CALD1 | ENSGALG00000013071 | 0.00054845 | down | Caldesmon/lymphocyte specific protein |
| MLCP | MBSP | ENSGALG00000010325 | 0.044965 | down | Ankyrin repeat-containing domain |
| Actin | ACTG2 | ENSGALG00000006343 | 0.038705 | down | Actin-related protein |
| ADORA2 | ADORA2B | ENSGALG00000014182 | 0.0042291 | up | 7TM GPCR, serpentine receptor class x |
| CRLR | RAMP1 | ENSGALG00000003821 | 0.00024318 | down | Receptor activity modifying protein |
| GS | GNAS | ENSGALG00000007509 | 0.0057521 | down | Guanine nucleotide binding protein (G-protein), alpha subunit |
| S-GC | GUCY1A3 | ENSGALG00000009354 | 1.86E-11 | down | Nucleotide cyclase |
| IRAG | MRVI1 | ENSGALG00000005632 | 0.023839 | down | inositol 1,4,5-triphosphate receptor-associated cGMP kinase substrate |
| **Calcium signaling pathway** | | | | | |
| GPCR | ADORA2B | ENSGALG00000014182 | 0.0042291 | up | GPCR, serpentine receptor class |
|  | ADRA1D | ENSGALG00000015974 | 2.67E-06 | down | G protein-coupled receptor, Adrenoceptor family |
|  | ADRA1B | ENSGALG00000001416 | 2.33E-05 | down | GPCR, rhodopsin-like |
|  | OXTR | ENSGALG00000003138 | 2.87E-07 | down | GPCR, rhodopsin-like |
| Gs | GNAS | ENSGALG00000007509 | 0.0057521 | down | Guanine nucleotide binding protein |
| PLCB | PLCB4 | ENSGALG00000008907 | 5.15E-05 | down | Phosphatidylinositol-4, 5-bisphosphate |
| CALM | CALM | ENSGALG00000010023 | 0.028664 | down | EF-hand, Ca insensitive |
| MLCK | MYLK | ENSGALG00000011708 | 8.22E-05 | down | myosin-light-chain kinase |
| CAMK | CAMK4 | ENSGALG00000000244 | 0.026825 | down | Serine/threonine/dual specificity protein kinase |
| ANT | SLC25A6 | ENSGALG00000016691 | 0.00018567 | down | Mitochondrial carrier protein |
| SPHK | SPHK1 | ENSGALG00000023541 | 0.026128 | up | Diacylglycerol kinase, catalytic domain |
| VADC | VDAC1 | ENSGALG00000006503 | 0.014133 | up | Porin, eukaryotic type |
| NOS | INOS | ENSGALG00000005693 | 3.54E-36 | up | Nitric oxide synthase |
| FAK2 | PTK2B | ENSGALG00000016564 | 0.0028046 | up | Serine-threonine/tyrosine-protein kinase catalytic domain |
| **TGF-β signaling pathway** | | | | | |
| BMP | BMP7 | ENSGALG00000007668 | 0.00706 | down | Transforming growth factor-beta |
| Smad6/7 | - | ENSGALG00000027843 | 7.30E-06 | down | SMAD/FHA domain |
| Skp1 | SKP1 | ENSGALG00000006474 | 0.031019 | down | SKP1 component, dimerisation |
| **Regulation of actin cytoskeleton** | | | | | |
| PI4P5K | PIP5K1B | ENSGALG00000015107 | 0.0026583 | down | Phosphatidylinositol-4-phosphate 5-kinase |
| MLCP | MBSP | ENSGALG00000010325 | 0.044965 | down | Ankyrin repeat-containing domain |
| MLCK | MYLK | ENSGALG00000011708 | 8.22E-05 | down | myosin light chain kinase |
| ITG | ITGA8 | ENSGALG00000008747 | 0.038479 | down | Integrin alpha chain |
| NHE1 | NHE1 | ENSGALG00000000921 | 0.01135 | up | Na+/H+ exchanger solute carrier family 9 |
| PI3K | PIK3R5 | ENSGALG00000021573 | 2.22E-05 | up | Phosphoinositide 3-kinase |
| NAP125 | NCKAP1L | ENSGALG00000027417 | 0.034648 | up | Nck-associated protein 1 |
| Arp2/3 | ARPC1B | ENSGALG00000022490 | 0.00079848 | up | actin related protein 2/3 complex |

**Note:** A gene with a Padj<0.05 is considered as significantly differential expressed. Padj means the corrected-P value. Up means this gene was up-regulated while down means this gene was down-regulated.
